# Neuroinflammatory Profiling in SIV-Infected Chinese-Origin Rhesus Macaques on Antiretroviral Therapy

**DOI:** 10.3390/v14010139

**Published:** 2022-01-13

**Authors:** Antonio Solis-Leal, Summer Siddiqui, Fei Wu, Mahesh Mohan, Wenhui Hu, Lara A. Doyle-Meyers, Jason P. Dufour, Binhua Ling

**Affiliations:** 1 Host-Pathogen Interaction Program, Texas Biomedical Research Institute, 8715 W Military Dr., San Antonio, TX 78227, USA; aleal@txbiomed.org (A.S.-L.); fwu@txbiomed.org (F.W.); mmohan@txbiomed.org (M.M.); 2 Tulane National Primate Research Center, Tulane University, Covington, LA 70433, USA; siqbal1@tulane.edu (S.S.); ldoyle@tulane.edu (L.A.D.-M.); jdufour@tulane.edu (J.P.D.); 3 Tulane Center for Aging, School of Medicine, Tulane University, New Orleans, LA 70112, USA; 4 Center for Metabolic Disease Research, Department of Pathology and Laboratory Medicine, Lewis Katz School of Medicine, Temple University, Philadelphia, PA 19122, USA; wenhui.hu@temple.edu; 5 Department of Microbiology and Immunology, School of Medicine, Tulane University, New Orleans, LA 70112, USA

**Keywords:** human immunodeficiency virus, simian immunodeficiency virus, central nervous system, reservoir, antiretroviral therapy, non-human primates, rhesus macaques, immune activation, neuroinflammation

## Abstract

The central nervous system (CNS) HIV reservoir is an obstacle to achieving an HIV cure. The basal ganglia harbor a higher frequency of SIV than other brain regions in the SIV-infected rhesus macaques of Chinese-origin (chRMs) even on suppressive combination antiretroviral therapy (ART). Since residual HIV/SIV reservoir is associated with inflammation, we characterized the neuroinflammation by gene expression and systemic levels of inflammatory molecules in healthy controls and SIV-infected chRMs with or without ART. CCL2, IL-6, and IFN-γ were significantly reduced in the cerebrospinal fluid (CSF) of animals receiving ART. Moreover, there was a correlation between levels of CCL2 in plasma and CSF, suggesting the potential use of plasma CCL2 as a neuroinflammation biomarker. With higher SIV frequency, the basal ganglia of untreated SIV-infected chRMs showed an upregulation of secreted phosphoprotein 1 (SPP1), which could be an indicator of ongoing neuroinflammation. While ART greatly reduced neuroinflammation in general, proinflammatory genes, such as IL-9, were still significantly upregulated. These results expand our understanding of neuroinflammation and signaling in SIV-infected chRMs on ART, an excellent model to study HIV/SIV persistence in the CNS.

## 1. Introduction

In the era of ART, HIV-induced neuronal injury and loss is a great concern for HIV patients as it causes cognitive, motor, and behavioral dysfunction. The morbidity of HIV infection has been deeply studied and characterized, originating the term HIV-associated neurocognitive disorder (HAND) [1]. The CNS HIV reservoir is one of the main causes of residual neuroimmune activation, which significantly contributes to HAND in individuals on ART. The eradication of HIV reservoirs remains the biggest obstacle to finding a cure, both for alleviating low-grade neuroimmune activation and for reducing systemic viral burden [2]. To achieve this goal, it is critical to examine neuroimmune activation and inflammatory signaling during HIV persistence in the context of viral suppression by ART [3]. In both humans and non-human primate models, HIV and SIV do not distribute evenly in different regions of the brain. The hippocampus and the basal ganglia are the two areas where higher amounts of viral RNA have been reported in ART naïve individuals [4,5,6,7], and the viral presence in these regions affects their activity and functionality [8,9]. Furthermore, HIV persists in different cell types such as perivascular macrophages, microglia, and also possibly, astrocytes and pericytes [10,11,12,13,14].

Although it is suspected that there is an association between viral persistence and the immune activation that triggers neuroinflammatory signaling [15,16], the degree of immune activation in the CNS in HIV patients on ART is still under debate [17]. There are several issues associated with the detection of HIV-associated neuroimmune activation and neuroinflammation, including limited biomarkers that can be used to reflect the CNS neuroinflammation [17,18]. Also, there is a lack of information regarding regional neuroimmune activation and neuroinflammation in areas of the brain with higher HIV persistence, even on suppressive ART. Finally, there is a low ART permeability through the blood-brain barrier (BBB) and a high active efflux from the CNS [19,20]. Even if the drugs penetrate the CNS at a desirable concentration, their use might result in CNS toxicity that causes pathogenic cellular and tissue damages [21].

In this context, the non-human primate (NHP) model could be the key to answering some questions regarding HIV CNS invasion, persistence, immune activation, and how these factors are affected by ART. This model has been used to study viral latency and persistency in the CNS [22]. In SIV infection of pigtailed macaques on ART, it was demonstrated that the CNS contains a stable SIV DNA reservoir and that animals display persistent CNS inflammatory responses regardless of ART. However, ART showed some capacity to reduce the CNS inflammatory responses [22]. Likewise, other groups showed that low BBB-penetrating antiretrovirals can reduce brain virus burden with short-term therapy in rhesus macaques, although they proposed that longer treatment may be required to diminish myeloid reservoirs [23]. More studies are needed regarding the modulation of pro-inflammatory signals that SIV^+^ macaques experience under ART, especially, given that HIV^+^ patients, even under ART, have shown signals of CSF immune activation [24]. One chemokine of special interest regarding HAND is the C-C Motif Chemokine Ligand 2 (CCL2, also called Monocyte chemoattractant protein-1, MCP-1). This pro-inflammatory molecule is a chemoattractant [25], which in the context of HIV infection, could enhance the infection of HIV targets expressing the CCR2 receptor and the formation of reservoirs. Extremely high expression of CCL2 as a result of SIV infection has been shown to predict SIV encephalitis [26], and therefore, is a key element to study to find therapies that can efficiently prevent HAND.

Previously, we used chRMs as an NHP model to study HIV reservoirs in the CNS. We examined 16 specific regions of the brain and 4 regions of the spinal cord. We found an uneven distribution of SIV in the brain with the highest frequency of SIV DNA in the basal ganglia [6]. Moreover, it is known that residual HIV/SIV reservoir is associated with varying degrees of neuroimmune activation [6]. Based on these results, we further tested the hypothesis that residual neuroimmune activation (enhanced proinflammatory gene/protein expression and signaling) in specific brain regions may potentially contribute to the pathogenesis of HAND. These findings could be remarkably important for further studies since regional neuroinflammatory signaling might precede observable inflammation and neuropathogenesis, especially in specific brain regions such as the basal ganglia where there are higher frequencies of SIV detection [6]. chRMs develops SIV-related diseases slower than Indian-origin RM (inRM) and, even on suppressive ART, including mild neuroinflammation in aged animals [27,28]. We used the antiretroviral drugs Tenofovir and Emtricitabine, which have shown CNS permeability [29].

Since residual neuroimmune activation in SIV-infected chRMs has not been well studied, we characterized the expression of proinflammatory signaling molecules in plasma, CSF, and basal ganglia of chRMs in SIV^+^ untreated, SIV^+^ ART-treated animals, and healthy chRMs. Additionally, we found a remarkable positive correlation between plasma and CSF levels of CCL2 which warrants further study to determine whether CNS inflammation levels could be inferred by simply testing CCL2 levels in peripheral blood. In addition, we tested the gene expression associated with the activation of key proinflammatory pathways in the basal ganglia caused by SIV infection and their modulation after ART.

## 2. Materials and Methods

### 2.1. Animals and SIV Infection

Animals were housed at the Tulane National Primate Research Center (TNPRC) and maintained following the standards of the American Association for Accreditation of Laboratory Animal Care and the “Guide for the Care and Use of Laboratory Animals” prepared by the National Research Council. All studies were approved by the Tulane Institutional Animal Care and Use Committee (IACUC). A total of 19 Rhesus macaques (*Macaca mulatta*) of Chinese origin were used in this study. They were divided into 3 groups, the SIV-naïve healthy group (control, *n* = 5), SIV-infected without ART (untreated, *n* = 7), and SIV-infected with ART (ART, *n* = 7). Animals were sera-negative for SIV, simian D retrovirus, and simian T-cell leukemia virus before SIV inoculation. Except for the healthy control group, the animals in the other two groups were inoculated intravenously with 100 TCID50 of SIVmac251.

### 2.2. Antiretroviral Therapy

Starting at 4 weeks post-infection, each animal received a daily subcutaneous injection containing an ART of reverse transcriptase inhibitors, composed of tenofovir, 20 mg/kg; and emtricitabine, 40 mg/kg. ART was continued until the end of the study, for a total of 24 weeks. Tenofovir and emtricitabine were generously provided by Gilead Sciences, Inc. (Foster City, CA, USA). Our previous report has shown the capacity of this drug combination to fully suppress viral replication in the SIV-infected chRM model [6].

### 2.3. Animal Euthanasia and Brain Tissue Collection

Experimental macaque groups were euthanized following the Tulane IACUC standards of operation and the AVMA Guidelines on the euthanasia of animals. Animals were anesthetized using telazol and buprenorphine, followed by a lethal intravenous injection of sodium pentobarbital. Fresh tissues from brain regions, such as the basal ganglia and frontal lobe, were collected and snap-frozen or stored in RNAlater during necropsies.

### 2.4. Cytokine/Chemokine Measurements in Plasma and CSF

Plasma from EDTA-treated blood and CSF were used for inflammatory cytokine and chemokine assay. Twenty-nine cytokines/chemokines were measured using the Monkey Cytokine Magnetic 29-plex multiplex immunoassay panel for the Luminex^TM^ platform according to the manufacturer’s instructions. (Thermo Fisher Scientific, Waltham, MA, USA).

### 2.5. PCR Array for Evaluation of Inflammatory Cytokine and Chemokine Gene Expression

The number of animals used in each group for this test were 5 in the control group, 3 in the untreated group, and 4 in the ART group. RNA was extracted from the basal ganglia preserved in RNAlater using the RNeasy microarray tissue RNA isolation kit (Qiagen). cDNA was then prepared with the Qiagen RT^2^ first-strand cDNA kit and amplified using the rhesus macaque inflammatory cytokines and receptors PCR array kit (Qiagen). Molecular levels of cytokines were compared between groups of the control, untreated, and ART. Fold change was calculated using the 2^-Delta Delta Ct formula using beta-actin, Beta-2-microglobulin, Glyceraldehyde-3-phosphate dehydrogenase, Hypoxanthine-guanine phosphoribosyltransferase-like and Ribosomal protein L13A as housekeeping genes for each sample. Further details of the results can be found in the Supplementary Materials File S1.

### 2.6. Gene Expression and Pathway Analysis

Additional bioinformatics analyses were performed using Ingenuity Pathway Analysis (IPA) (QIAGEN Inc., Hilden, Germany. https://www.qiagenbioinformatics.com/products/ingenuity-pathway-analysis), last accessed date 10 January 2022 [30]. Using the PCR array results, differentially expressed genes (DEGs) were defined by fold change > 2 and *p*-value < 0.05. Then, we conducted canonical pathways enrichment, biological function annotations, and regulator effect analysis using these DEGs.

### 2.7. Statistical Analysis

Non-parametric Mann-Whitney tests were used to compare cytokine/chemokine levels in plasma and CSF between groups of the control, untreated, and ART animals. The Spearman correlation was used to assess the correlation of cytokine and chemokine levels between paired plasma and CSF samples. GraphPad Prism 9.0.1 statistical software (GraphPad Software, Inc., San Diego, CA, USA) was used to analyze data, and statistical results were set to two-sided at *p* < 0.05 as significant.

## 3. Results

### 3.1. CCL2 and IFN-γ Levels Were Significantly Elevated in Peripheral Blood of SIV-Infected chRMs Receiving Suppressive ART

**Figure 1:**
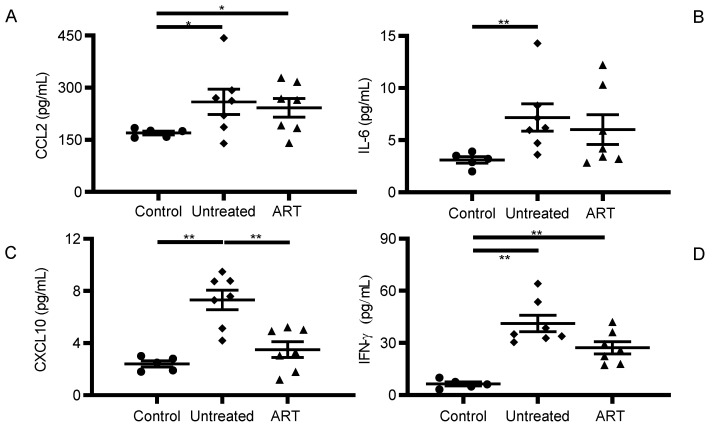
Differential cytokine and chemokine levels in plasma of the different groups of chRMs. The scatter graphs show the distribution observed in the plasma of each group for the cytokines and chemokines: (**A**) CCL2, (**B**) IL-6, (**C**) CXCL10, and (**D**) IFN-g. (* *p* < 0.05, ** *p* < 0.01).

In this study, we tested the levels of 29 pro-inflammatory cytokines and chemokines in plasma samples of ART naïve and treated SIV-infected chRMs. Most of cytokines/chemokines were undetectable or did not show a significant difference between the untreated and the ART group. We paid special attention to CCL2, IL-6, CXCL10, and IFN-γ plasma levels, since they have been reported to be dysregulated during ART in HIV and SIV infection [31,32]. The levels of each were significantly increased under SIV infection. However, although the ART showed a general tendency to decrease levels of cytokines/chemokines, only CXCL10 showed a significant reduction by ART compared to the untreated group (Figure 1).

CCL2 levels were statistically higher in the untreated (259.2 ± 96.62 pg/mL) (mean ± sd) compared to the control group (169.9.3 ± 12.08 pg/mL, *p* = 0.048). Interestingly, plasma CCL2 levels in the ART group remained elevated and were comparable to the untreated group. The levels were also significantly higher than the control group (242.1 ± 71.13 pg/mL, *p* = 0.048).

IL-6 levels were only statistically different when comparing the untreated (7.17 ± 3.49 pg/mL, *p* = 0.005) and the control group (3.1 ± 0.72 pg/mL). Although slightly increased, no statistical difference was found between the ART and the control group (6.402 ± 3.76 pg/mL, Figure 1B).

CXCL10 levels were overall low, with 2.40 ± 0.53 pg/mL in the control group, 7.31 ± 1.98 pg/mL in the untreated group, and 3.50 ± 1.62 pg/mL in the ART group (Figure 1C). Interestingly, this cytokine displayed a statistically significant increase due to SIV infection that was successfully counteracted by the effect of ART (*p* values 0.003 and 0.007 respectively).

IFN-γ levels showed a similar pattern to CCL2. Plasma levels of IFN-γ were 6.38 ± 2.60 pg/mL in the control group, 41.21 ± 12.70 pg/mL in the untreated group, and 27.25 ± 9.29 pg/mL in the ART group. Again, SIV infection without treatment showed a statistically significant increase (*p* = 0.003) and ART failed to reduce it to the levels detected in the control group (*p* = 0.003, Figure 1D).

The overall decreased levels of these pro-inflammatory markers in plasma suggest a positive effect of ART in alleviating systemic inflammation in chRMs. Nevertheless, the effect of ART was not potent enough to decrease CCL2 and IFN-γ to preinfection levels, showing some limitations to completely revert immune activation.

### 3.2. ART Reduced Inflammatory Cytokine and Chemokine Levels in CSF of SIV-Infected chRMs

**Figure 2:**
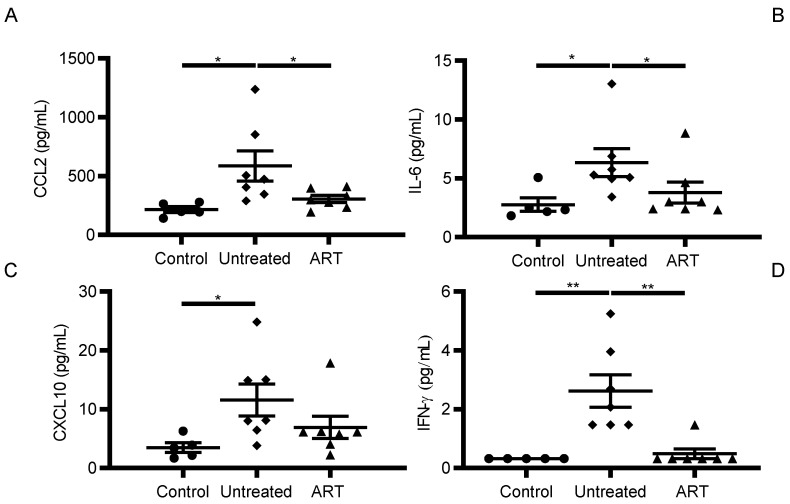
Differential cytokine and chemokine levels in CSF of chRMs. The scatter graphs show the distribution observed in the CSF of the experimental groups for the cytokines (**A**) CCL2, (**B**) IL-6, (**C**) CXCL10, and (**D**) IFN-g. (* *p* < 0.05, ** *p* < 0.01). Non-parametric Mann-Whitney tests.

The same panel of 29 cytokines and chemokines was next quantitated in the CSF of the same group of SIV-infected chRMs. Again, since many of them remain stable during SIV infection, we focused on CCL2, IL-6, CXCL10, and IFN-γ. Similar to the trend seen in plasma, pro-inflammatory cytokine levels in the CSF were increased after SIV infection but levels of all four were successfully decreased by ART. In particular, CSF levels of CCL2 and IFN-γ, showed a statistically significant reduction in the ART compared to the control group (Figure 2).

SIV infection caused significantly high levels of CCL2 (587.4 ± 339.6 pg/mL in the untreated group) compared to the control group (214.8 ± 55.86 pg/mL, *p* = 0.025), and ART effectively lowered CCL2 (306 ± 81.17 pg/mL, *p* = 0.0262) to levels comparable to the uninfected control group (Figure 2A).

Likewise, SIV infection significantly increased IL-6 levels (6.34 ± 3.12 pg/mL) compared to the control group (2.77 ± 1.31 pg/mL, *p* = 0.011) that was significantly reduced by ART compared to the untreated group (3.81 ± 2.36 pg/mL, *p* = 0.022). Overall, IL-6 levels in the ART group was comparable to the control group (Figure 2B).

CXCL10 levels were relatively low in the CSF with 3.47 ± 1.81 pg/mL in the control group, 11.58 ± 7.17 pg/mL in the untreated group, and 6.92 ± 5.05 pg/mL in the ART group. SIV infection resulted in a statistically significant (*p* = 0.01, Figure 2C) increase in CXCL10 levels.

IFN-γ levels were below the limit of detection in the control group, 2.62 ± 1.47 pg/mL in the untreated group, and 0.48 ± 0.44 pg/mL in the ART group. SIV infection led to a statistically significant increase in IFN-γ levels (*p* = 0.001) and ART significantly (*p* = 0.002) lowered it to the levels detected in the uninfected group (Figure 2D). Taken together, the CSF showed a decrease in the levels of pro-inflammatory markers after ART.

### 3.3. CCL2 Linear Correlation between CSF and Plasma Levels

CSF collection permits a minimally invasive approach to detect surrogate biomarkers of CNS inflammation longitudinally during HIV/SIV infection to evaluate the effectiveness of cART on neuroinflammation. However, its acquisition can still be challenging in the clinic due to the invasive techniques required to obtain it.

**Figure 3:**
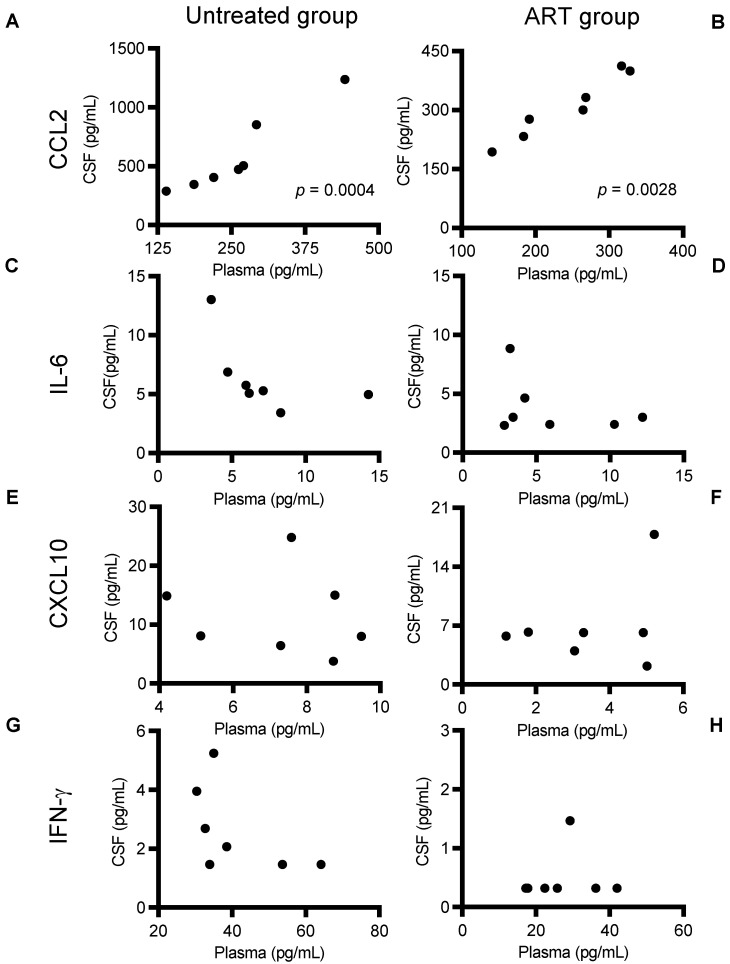
Association of levels of cytokine and chemokine between the CSF and plasma of SIV-infected chRMs with ART (**right** panels) or without ART (**left** panels). (**A**,**B**), (**C**,**D**), (**E**,**F**) and (**G**,**H**) show CCL2, IL-6, CXCL10, and IFN-γ levels respectively.

To study whether inflammation markers in plasma predict or reflect changes in the brain, we analyzed correlations of the tested cytokines and chemokines (CCL2, IL-6, CXCL10, and IFN-γ) in paired plasma and CSF samples in each infected group separately (Figure 3). Very interestingly, CCL2, both in the untreated and the ART group, showed a strong positive correlation between plasma and CSF levels (Figure 3A, *p* = 0.0004 and, *p* = 0.0028 respectively).

**Figure 4:**
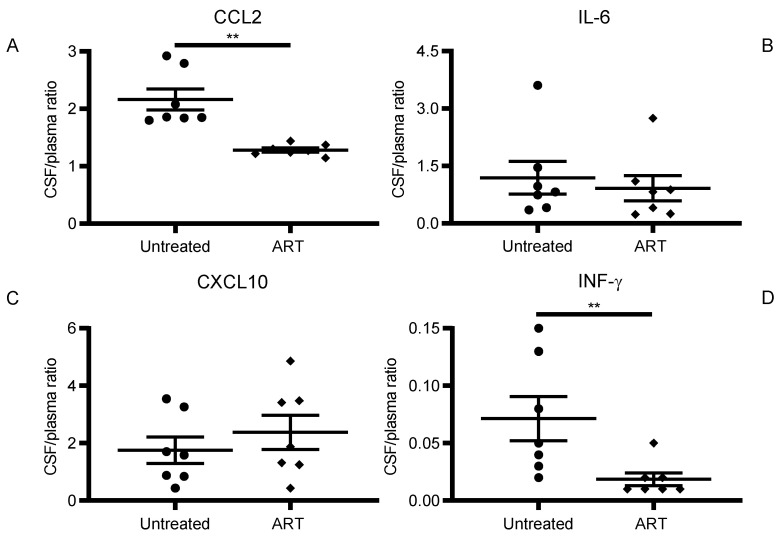
CSF/Plasma ratio difference between Untreated and ART groups. The graphs show the distribution observed in the CSF/Plasma ratio of the experimental groups for (**A**) CCL2, (**B**) IL-6, (**C**) CXCL10, and (**D**) IFN-γ (** *p* < 0.01).

There was no significant correlation between levels in the peripheral blood and CSF for the rest of the cytokines and chemokines studied in the untreated and ART groups. IL-6 (C,D), CXCL10 (E,F), and IFN-γ (G,H). Overall, only CSF/plasma values for CCL2 showed a positive correlation. Levels between CSF and plasma showed a decrease in ratio for the ART groups for both CCL2 and IFN-γ (Figure 4A,C), while ratios for IL-6 and CXCL10 remained similar for both groups.

### 3.4. Expression of Proinflammatory Cytokine and Chemokine Genes Remained Elevated in Basal Ganglia despite Long-Term Suppressive ART

Previously, CSF and plasma samples allowed us to determine the correlation that existed between cytokine and chemokine production. However, the activation of pro-inflammatory pathways in specific brain structures that contribute to the CSF profile remains to be defined. In an earlier study, we demonstrated that SIV DNA was detected in a broad range of brain regions even in animals on long-term ART [6]. In this study, we specifically focused on the basal ganglia since it is one of the major HIV/SIV targets in the brain with the highest viral loads found in brain tissues [33]. Therefore, we further studied gene expression profiles related to proinflammatory signaling in this region in the different chRM groups.

**Figure 5:**
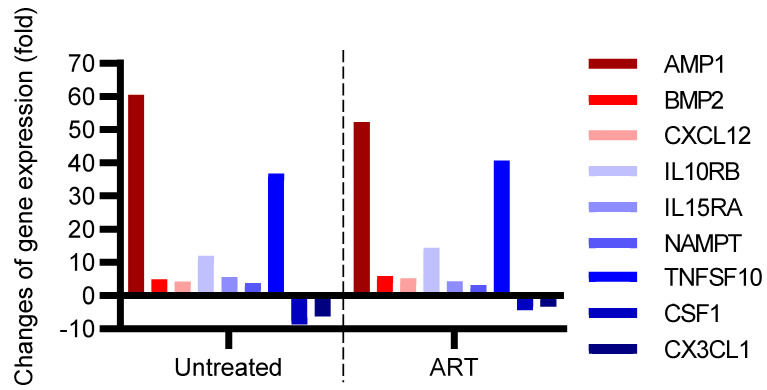
Profiling of gene expression in the basal ganglia of SIV^+^ chRMs with antiretroviral therapy (ART) or without ART (Untreated). The fold-changes of gene expression in the ART and Untreated groups were shown separately in comparison with the healthy control group.

Eighty-four genes were tested. Gene expression with significant (*p* < 0.05) fold changes compared to the control group is shown in Figure 5. Seven genes were significantly (*p* < 0.05) upregulated in both the untreated and ART group: *AIMP1*, *BMP2*, *CXCL12*, *IL-10RB*, *IL-15RA*, *NAMPT*, and *TNFSF10* (Table 1). Three of these genes had more than a 10-fold increase in both groups. *AIMP1*, (Aminoacyl tRNA synthetase complex-interacting multifunctional protein 1), which has been linked to the secretion of CCL2 by macrophages and monocytes [34], showed a 61-fold expression increase in the untreated group, and a 52-fold increase in the ART group. *IL-10RB* (Interleukin-10 receptor subunit β-like), which is related to antiviral activity and is the receptor of the anti-inflammatory cytokine IL-10 [35,36], showed a 12-fold increase in the untreated group, and a 14-fold increase in the ART group (Table 1). *TNFSF10*, tumor necrosis factor ligand superfamily member 10, related to apoptosis and inflammation [37], was 37-fold higher in the untreated group and even higher in the ART group with a 41-fold change (Table 1). Only 2 genes, namely, *CSF1*, colony-stimulating factor 1 (macrophage), and *CX3CL1* (C-X3-C motif chemokine ligand 1) were downregulated in both groups. Interestingly, CCL2 and IL-6, which showed high expression in the CSF showed no significant changes at the gene expression level in the basal ganglia in either group.

### 3.5. Expression of Proinflammatory IL-11RA, IL-16, IL-6R, and IL-9 Remained Elevated in the Basal Ganglia despite Receiving Long-Term Suppressive ART

**Figure 6:**
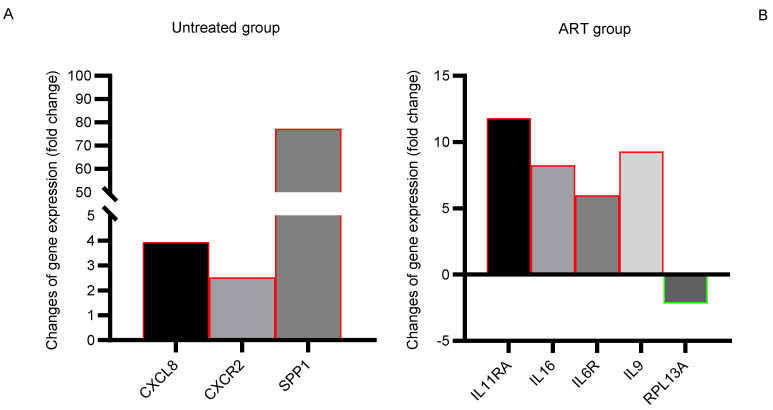
Gene expression changes in the basal ganglia found exclusively in either the Untreated group or the ART group, compared to the healthy control. (**A**) Genes upregulated only in the Un treated group compared to the healthy control group. (**B**) Genes up/downregulated only in the ART group compared to the healthy control group. Red or green borders in the bar graphs represent upregulation or downregulation respectively compared to the control group.

In addition to the previous section showing genes that were parallelly up/down-regulated in basal ganglia in both untreated and ART groups, Figure 6 shows genes that were differentially modulated only in one of the two groups compared to the control group. SPP1 (secreted phosphoprotein 1, also known as osteopontin, OPN), associated with neuroinflammation in macaques [38,39], was strikingly increased by 77-fold in the untreated group. In addition, CXCL8 and CXCR2 were upregulated with a 3- and 4-fold increase in untreated animals but not in the ART group (Figure 6A, Table 1). In contrast, the ART group showed an upregulation of the genes IL-11RA, IL-16, IL-6R, and IL-9 in a range of 8~12-fold. In contrast, RPL13A was slightly (2-fold) downregulated compared to the control group (Figure 6B, Table 1).

### 3.6. Functional Analysis of Differentially Expressed Genes in SIV^+^ Untreated and ART Animals

**Figure 7:**
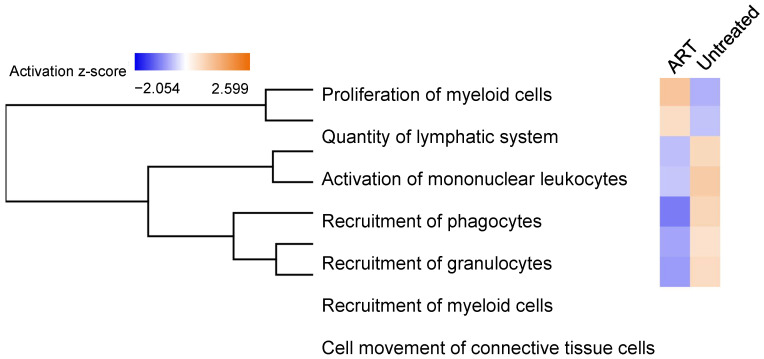
Biological Functional Annotations in Untreated vs. ART. DEGs in the ART and the untreated groups were analyzed. Differential pro-inflammatory functions were enriched for each group. The activation and inhibition of functions are shown by orange and blue colors, respectively. DEGs were defined by fold change > 2 and *p* < 0.05.

**Figure 8:**
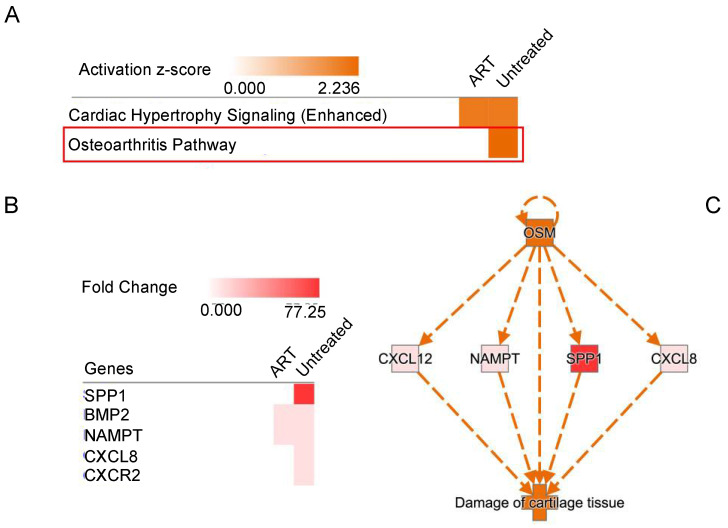
ART vs. Untreated Pathway Analyses. (**A**) Canonical pathways enrichment analysis. The red box highlights the discrepant pathway between groups. (**B**) Expression of genes in the osteoarthritis pathway. (**C**) Regulator Effects network in the untreated group. It illustrates the relationships between the upstream regulator (OSM) and downstream function and diseases (damage of cartilage tissue). The measured and predicted activation is represented by the red and orange colors, respectively.

Further analysis of functional changes in the two animal groups allowed for characterization and identification of gene expression patterns. The untreated group had genes enriched for functional groups associated with the activation of mononuclear leukocytes, recruitment of phagocytes, recruitment of granulocytes, recruitment of myeloid cells, and cell movement/migration of connective tissue cells. These genes were downregulated in the ART group. Interestingly, the proliferation of myeloid cells and quantity of lymphatic system were downregulated in the untreated group but upregulated in the ART group (Figure 7). In addition, with the extremely high SPP1 expression in the basal ganglia in the untreated animals, the SPP1 regulatory pathway and other genes such as CXCL8 were found to be integral to the Oncostatin M (OSM) network (Figure 8).

## 4. Discussion

HIV and SIV infection lead to CNS inflammation and the degree of severity is dictated by different factors, such as plasma and CSF viral loads or the stage of HIV/SIV infection [40]. Pro-inflammatory cytokines and chemokines are usually upregulated and reported to drive CNS disease progression. It has been reported that HIV-infected individuals on suppressive ART continue to have high circulating levels of pro-inflammatory cytokines, which helps to maintain a heightened proinflammatory state [41]. In this study, we observed that ART significantly reduced levels of some proinflammatory cytokines in peripheral blood and CSF of SIV-infected chRMs (Figure 1 and Figure 2), while others were not affected by the treatment. Although twenty-nine cytokines and chemokines were tested in plasma and CSF samples, CCL2, IL-6, CXCL10, and IFN-γ showed significant changes in the CSF during chronic SIV infection with or without ART.

CCL2 is a pro-inflammatory chemokine with the ability to enhance viral replication and pathogenesis. Both HIV and its viral products have been found to enhance the expression of this chemokine in HIV patients, being closely related to the immune activation and inflammation observed in patients even under ART [42]. Therefore, the upregulation observed in the untreated group both for plasma and CSF samples was highly expected (Figure 1A and Figure 2A). IL-6 is a molecule produced in response to infections and tissue injury [43]. Its upregulation has been associated with HIV infection regardless of ART treatment in HIV patients [44]. Therefore, its increased levels observed even under ART suggests that the chRM model mirrors the findings made on IL-6 levels reported in HIV patients (Figure 1B and Figure 2B). CXCL10 (C–X–C motif chemokine 10) is also known as Interferon γ-induced protein and induces chemotaxis of monocytes and microglia [45]. In the context of HIV infection, CXCL10 produced by Th1 cells has been associated with HIV replication. High levels of this chemokine in plasma during early infection is a hallmark of rapid AIDS progression [46]. IFN-γ is a pleiotropic cytokine released by T-lymphocytes and natural killer cells. Usually, these cells do not cross the BBB at appreciable levels and, as such, IFN-γ is generally undetectable within the central nervous system (CNS) [47].

These results suggest a possible use of the plasma CCL2 levels as a biomarker that reflects CCL2 levels in the brain to indirectly monitor its overall inflammatory status (Figure 3A,B). Considering that the ratio of CCL2 is significantly different in the presence of ART (Figure 4A), this correlation is even more meaningful in the context of SIV infection and potentially HIV infection under ART. This is especially relevant in the era of ART, where this parameter could be used to predict persistent low-grade neuroimmune activation if baseline CCL2 levels are also measured. Nevertheless, it is worthy to note that further studies are needed to determine if this association could change under different experimental conditions in animals or different ART combinations in humans. Additionally, we were not able to identify the cause of the upregulation of CCL2 in plasma. Some studies have described how CCL2/CCR2 and CXCL10 is upregulated in gut tissues in the context of HIV infection, potentially contributing to the dysregulation observed [48,49]. Moreover, the lack of CCL2 downregulation in plasma after ART treatment suggests that this correlation may change in the context of ART. Since CCL2 plasma levels in blood after ART may be reduced at a slower rate than the CCL2 levels in the CSF.

The current cytokine and chemokine focused neuro AIDS study shows that the chRM model, regardless of the presence or absence of encephalitis, displayed a pro-inflammatory response in the brain similar to that observed in HIV patients, demonstrating the usefulness of this model to understand neuroimmune activation and the pro-inflammatory response in HIV-infected individuals.

Gene expression was then profiled to understand whether the basal ganglia contribute to the increase in CSF cytokine and chemokine levels. Additional genes were included in Figure 5 that showed a fold change between 1 and 10 that were similarly modulated in the untreated and the ART group (Table 1). This included *BMP2* (bone morphogenetic protein-2), which is a potent osteoinductive cytokine and is also linked to inflammation and leukocyte recruitment [50,51]; *CXCL12* is a pro-inflammatory chemokine that plays a role in maintaining the homeostasis in the adult brain and has been reported to be altered in cases of HIV-1-associated encephalopathy [52]; *IL-15RA* is a receptor of the pro-inflammatory cytokine IL-15 that promotes activation, proliferation, and survival of T-lymphocytes and natural killer cells [53]; and *NAMPT* (Nicotinamide phosphoribosyltransferase), with a newly discovered function as an extracellular endogenous mediators of inflammation [54]. The downregulated gene *CSF1* (Colony-stimulating factor) is related to the differentiation of macrophages and microglia [55,56]. Conversely, another group reported an upregulation of *CSF1* in the SIV RM model. However, that study was performed in the frontal cortex of pigtailed macaques but not in chRMs, suggesting that the different regions of the brain studied and the different species used may explain the discrepancy [57]. CX3CL1 together with Fractalkine forms a transmembrane structure that serves both as an adhesion molecule and as a chemoattractant [58]. In the brain, this chemokine is primarily expressed in neurons, and its receptor (CX3CR1) is mainly expressed in microglia, establishing an interaction between them [59,60]. HIV-1 Tat protein has been reported to cause downregulation of *CX3CR1* in microglia, inducing pro-inflammatory responses [61]. Similarly, we observed a downregulation of *CX3CL1*, which may be driven by the presence of viral protein and the inflammation status previously reported. The lack of direct CCL2 modulation by the basal ganglia suggests that other brain tissues/structures might contribute to the CCL2 CSF downregulation observed in the ART group (Figure 2A).

Moreover, regardless of ART treatment SIV is still able to downregulate CSF1 and CX3CL1 in both experimental groups. Some reports have shown that brain macrophages and microglia upregulate CSF1/CSF1R under SIV/HIV infection [57,62,63]. However, the similar pattern observed in these two experimental groups suggests that the expression of both genes is generally downregulated in the basal ganglia.

While the genes discussed above were parallelly-modulated in the basal ganglia for both groups of SIV^+^ untreated and ART, unique genes differentially expressed in either group were also found. The untreated group (Figure 6A) showed the upregulation of *SPP1*, *CXCL8*, and *CXCR2*. These genes are associated with inflammation [38,39,64], with both *CXCL8* and *CXCR2* intimately related to the regulation of the BBB [65]. The dysfunction of the BBB is suspected to be the root cause of several neurocognitive disorders, such as multiple sclerosis, Alzheimer’s disease, and HAND [66,67]. This remarkable upregulation of pro-inflammatory genes might be linked to the high viral replication in the basal ganglia in untreated SIV infection, thus, enhancing local neuroinflammation [4]. On the other hand, the upregulated genes *IL-11RA* and *IL-9* in the ART group (Figure 6B) have shown an anti-inflammatory effect on the CNS [68,69,70]. However, *IL-16* has been described as a pro-inflammatory gene [71]. Conversely, even though *IL-6R* has been classically referred to as a pro-inflammatory gene, recent papers describe additional anti-inflammatory roles of this cytokine [43,72]. Therefore, this upregulation could either be linked to the global anti-inflammatory effect of ART (supposing an anti-inflammatory role) or be related to ART CNS toxicity (assuming a pro-inflammatory role) [21]. *RPL13A* (Ribosomal protein L13a), downregulated in the ART group is a pro-inflammatory protein that engages the IFN-γ-mediated inflammatory response to modulate gene expression [73]. Figure 6 adds on the anti-inflammatory effect of ART, showing the downregulation of pro-inflammatory genes or the upregulation of anti-inflammatory genes. Conversely, the pro-inflammatory genes *IL-16*, *BMP2*, *CXCL12*, and *TNFS10* were upregulated by ART, suggesting the persistence of neuroinflammation during suppressive ART.

Lastly, Figure 7 shows how SIV enhances the production of recruitment molecules, which could increase infiltration of pro-inflammatory cells into the CNS of chRMs, that was alleviated by ART. In addition, the study of regulatory genes allowed the identification of genes related to cartilage damage, especially in the untreated group (Figure 8A,B) suggesting that the SSP1-mediated proinflammatory pathway is common to arthritis and neuroinflammatory diseases like HAND. OSM belongs to the IL-6 family and has been demonstrated to become upregulated in pathological conditions, activating the JAK-STAT and MAPK pathways which may lead to neuroinflammation [74]. More studies need to be conducted in the brains of animal models to understand how activation of the OSM pathway may affect the functionality of certain areas and whether it is correlated to neuroinflammation and HAND. Further investigation of this correlation may be particularly relevant in developing countries where there is limited access to ART since this upregulation could be dramatically detrimental in the absence of treatment (Figure 6A and Figure 8C, Table 1).

## 5. Conclusions

The present study has shown that ART may alleviate neuroinflammation in SIV-infected chRMs by modulating the levels and expression of some inflammation-related genes and neuroimmune activation pathways. On the other hand, ART itself may also cause dysregulation of some other neuroinflammatory signaling. To our knowledge, this is the first study to investigate a wide range of inflammatory cytokines, chemokines, and genes modulated by SIV and ART in the chRM model. Nevertheless, we were unable to monitor the untreated SIV^+^ chRMs to the end stage of AIDS to confirm if some of them eventually develop encephalitis with presence of significant histopathologic lesions in the brain, which could directly correlate with significantly increased pro-inflammatory markers in the CSF and brain regions. In addition, we were unable to identify the causality of CCL2 correlation between the CSF and plasma. Further research could be conducted in other tissues, both from different brain regions and from different body tissues, to identify the root cause and degree of the CCL2 upregulation.

In conclusion, these observations emphasize the potential use of plasma CCL2 levels as a biomarker to extrapolate CSF levels given the correlation explained above. This finding is novel and could be highly useful in clinical diagnosis, as the findings propose a less invasive technique that could allow the detection of neuroinflammation without having to collect CSF in HIV patients on/off ART. Future studies are needed to further evaluate its feasibility and usefulness in HIV patients on ART. Our findings identify chRMs as an excellent NHP model to study HAND that is frequently found as the consequence of HIV-driven residual CNS immune activation and inflammation that persists under suppressive ART. Finally, despite the finding that ART partially aids to alleviate neuroimmune activation, ART drugs (one or both) might also potentially drive the dysregulation of some genes relevant to neuroinflammation.

## Figures and Tables

**Table 1:** Gene expression fold change and *p*-value for each experimental group against the control group.

Gene	Fold Change in Untreated Group *vs.* Control Group	*p*-Value	Gene	Fold Change in ART Group *vs.* Control Group	*p*-Value
*AIMP1*	60.62	0.0006	*AIMP1*	52.35	0.0003
*BMP2*	4.90	0.0138	*BMP2*	5.85	0.0184
*CXCL12*	4.23	0.0380	*CXCL12*	5.17	0.0021
*IL10RB*	11.93	0.0014	*IL10RB*	14.35	0.0054
*IL15RA*	5.58	0.0021	*IL15RA*	4.24	0.0219
*NAMPT*	3.72	0.0039	*NAMPT*	3.20	0.0145
*TNFSF10*	36.72	0.0003	*TNFSF10*	40.67	0.0004
*CSF1*	−8.58	0.0006	*CSF1*	−4.28	0.0010
*CX3CL1*	−6.26	0.0093	*CX3CL1*	−3.28	0.0087
*CXCL8*	3.93	0.0057	*IL11RA*	11.79	0.0208
*CXCR2*	2.51	0.0426	*IL16*	8.23	0.0035
*SPP1*	77.25	0.0165	*IL6R*	5.96	0.0010
			*IL9*	9.26	0.0106
			*RPL13A*	−2.20	0.0389

Note: Fold-change values greater than one indicate up-regulation. Fold-change values lower than one indicate down-regulation. The grey area represents genes found individually modulated in either the Untreated or ART group, which were shown in Figure 6.

## Supplementary Materials

The following supporting information can be downloaded at: https://www.mdpi.com/article/10.3390/v14010139/s1, File S1: RT² Profiler PCR Array Gene Expression Analysis Report.
