# Supplementary material for: Neuroinflammatory Profiling in SIV-Infected Chinese-Origin Rhesus Macaques on Antiretroviral Therapy
Source: Viruses. 2022 Jan 13;14(1):139. doi: 10.3390/v14010139 (PMC8781366; doi:10.3390/v14010139)
Supplement: Supplementary file 1 [file viruses-14-00139-s001.zip › viruses-1534119-supplementary.pdf]

QIAGEN

RT² Profiler PCR Array Gene Expression Analysis Report

Summer Iqbal

Wednesday, June 26, 2019

Table of Contents

Introduction 1
Summary and workflow 2
Gene table 3
Data analysis setup 5
Data quality control (QC) 6
Normalization analysis 7
Results 8
Fold regulation comparison and p-value 8
Scatter Plot 10
Volcano Plot 16
Heat Map 22
ClusterGram 24
What's next? 25
Gene Expression 27
DNA Methylation 27
RNAi 27
Somatic Mutation 27
miRNA Regulation 29
Transcription Factor / Histone 33
Next steps 35
Glossary 36

Introduction

Cataloged arrays

RT^2 Profiler PCR Arrays are highly reliable and sensitive gene expression profiling tools for analyzing focused panels of genes in signal transduction, biological processes or disease research pathways using real-time PCR. Each cataloged RT^2 Profiler PCR Array contains a list of the pathway-focused genes as well as five housekeeping (reference) genes on the array. In addition, each array contains a panel of proprietary controls to monitor genomic DNA contamination (GDC) as well as the first strand synthesis (RTC) and real-time PCR efficiency (PPC). The qPCR Assays used in PCR Arrays are laboratory-verified and optimized to work under standard conditions enabling a large number of genes to be assayed simultaneously. Their specificity is guaranteed when RT^2 SYBR Green qPCR Master Mixes are used as part of the complete PCR Array System protocol.

In this study, 96 genes were profiled on 12 samples with the PAQQ-011Z.

Summary and workflow

Cataloged arrays

1. Mature RNA was isolated using an RNA extraction kit according to the manufacturer's instructions.
2. RNA quality was determined using a spectrophotometer and was reverse transcribed using a cDNA conversion kit.
3. The cDNA was used on the real-time RT² Profiler PCR Array (QIAGEN, Cat. no. PAQQ-011Z) in combination with RT² SYBR® Green qPCR Mastermix (Cat. no. 330529).

C_T values were exported to an Excel file to create a table of C_T values. This table was then uploaded on to the data analysis web portal at **http://www.qiagen.com/geneglobe**. Samples were assigned to controls and test groups. C_T values were normalized based on a/an Manual Selection of reference genes.

The data analysis web portal calculates fold change/regulation using delta delta C_T method, in which delta C_T is calculated between gene of interest (GOI) and an average of reference genes (HKG), followed by delta-delta C_T calculations (delta C_T (Test Group)-delta C_T (Control Group)). Fold Change is then calculated using 2^ (-delta delta C_T) formula. The data analysis web portal also plots scatter plot, volcano plot, clustergram, and heat map.

This data analysis report was exported from the QIAGEN web portal at GeneGlobe.

Gene table

RT² Profiler™ PCR Array Rhesus Macaque Inflammatory Cytokines & Receptors

Position	RefSeq Number	Symbol	Description
A01	XM_001083775	AIMP1	Aminoacyl tRNA synthetase complex-interacting multifunctional protein 1
A02	XM_001115987	BMP2	Bone morphogenetic protein 2
A03	NM_001032849	CCL1	Chemokine (C-C motif) ligand 1
A04	NM_001032874	CCL11	Chemokine (C-C motif) ligand 11
A05	XM_001113462	CCL13	Chemokine (C-C motif) ligand 13
A06	NM_001032852	CCL17	Chemokine (C-C motif) ligand 17
A07	NM_001032821	CCL2	Chemokine (C-C motif) ligand 2
A08	NM_001032854	CCL20	Chemokine (C-C motif) ligand 20
A09	NM_001032946	CCL23	Chemokine (C-C motif) ligand 23
A10	NM_001032850	CCL5	Chemokine (C-C motif) ligand 5
A11	NM_001032858	CCR1	Chemokine (C-C motif) receptor 1
A12	NM_001047140	CCR3	Chemokine (C-C motif) receptor 3
B01	XM_001098807	CCR4	Chemokine (C-C motif) receptor 4
B02	NM_001032935	CCR6	Chemokine (C-C motif) receptor 6
B03	XM_001084047	CCR8	Chemokine (C-C motif) receptor 8
B04	NM_001032839	CD40LG	CD40 ligand
B05	XM_001088935	CD70	CD70 antigen-like
B06	XM_001090841	CSF1	Colony stimulating factor 1 (macrophage)
B07	NM_001032949	CSF2	Colony stimulating factor 2 (granulocyte-macrophage)
B08	NM_001032881	CX3CL1	Chemokine (C-X3-C motif) ligand 1
B09	XM_001084526	CX3CR1	Chemokine (C-X3-C motif) receptor 1
B10	NM_001032950	CXCL11	Chemokine (C-X-C motif) ligand 11
B11	NM_001032934	CXCL12	Chemokine (C-X-C motif) ligand 12
B12	NM_001032880	CXCL13	Chemokine (C-X-C motif) ligand 13
C01	XM_001103136	CXCL6	Chemokine (C-X-C motif) ligand 6 (granulocyte chemotactic protein 2)
C02	NM_001032936	CXCL9	Chemokine (C-X-C motif) ligand 9
C03	NM_001040420	CXCR1	Chemokine (C-X-C motif) receptor 1
C04	NM_001042645	CXCR4	Chemokine (C-X-C motif) receptor 4
C05	NM_001032838	FASLG	Fas ligand (TNF superfamily, member 6)
C06	NM_001135794	IFNA2	Interferon, alpha 2
C07	NM_001032905	IFNG	Interferon-gamma
C08	XM_001092736	IL10RA	Interleukin 10 receptor, alpha
C09	XM_001094340	IL10RB	Interleukin-10 receptor subunit beta-like
C10	XM_001093448	IL11RA	Interleukin 11 receptor, alpha
C11	NM_001044725	IL12B	Interleukin 12B (natural killer cell stimulatory factor 2, cytotoxic lymphocyte maturation factor 2, p40)
C12	XM_001115186	IL12RB1	Interleukin 12 receptor, beta 1
D01	XM_001094235	IL12RB2	Interleukin 12 receptor, beta 2
D02	NM_001032929	IL13	Interleukin 13
D03	NM_001044731	IL15	Interleukin 15
D04	NM_001079517	IL15RA	Interleukin 15 receptor, alpha
D05	NM_001032808	IL16	Interleukin 16
D06	XM_001106391	IL17A	Interleukin 17A
D07	XM_001106517	IL17F	Interleukin-17F-like
D08	NM_001042757	IL1A	Interleukin 1, alpha
D09	NM_001042756	IL1B	Interleukin 1, beta
D10	XM_001107510	IL1R1	Interleukin 1 receptor, type I

Position	RefSeq Number	Symbol	Description
D11	NM_001047134	IL1R2	Interleukin 1 receptor, type II
D12	XM_001091717	IL1RN	Interleukin 1 receptor antagonist
E01	NM_001047130	IL2	Interleukin 2
E02	XM_001084726	IL20	Interleukin 20
E03	XM_001103613	IL21	Interleukin 21
E04	NM_001032917	IL2RA	Interleukin 2 receptor, alpha
E05	XM_001085792	IL2RB	Interleukin 2 receptor, beta
E06	NM_001035529	IL2RG	Interleukin 2 receptor, gamma
E07	NM_001101734	IL3	Interleukin 3 (colony-stimulating factor, multiple)
E08	NM_001032904	IL4	Interleukin 4
E09	NM_001047133	IL5	Interleukin 5 (colony-stimulating factor, eosinophil)
E10	XM_001100843	IL5RA	Interleukin 5 receptor, alpha
E11	NM_001042733	IL6	Interleukin 6 (interferon, beta 2)
E12	XM_001114404	IL6R	Interleukin 6 receptor
F01	NM_001032846	IL7	Interleukin 7
F02	NM_001032965	CXCL8	Interleukin 8
F03	XM_015111045	CXCR2	Chemokine (C-X-C motif) receptor 2
F04	XM_001100521	IL9R	Interleukin 9 receptor
F05	XM_001102628	IL13RA2	Interleukin-13 receptor subunit alpha-2-like
F06	XM_001099282	LOC71061 8	Hypothetical LOC710618
F07	XM_001110897	IL9	Interleukin-9-like
F08	XM_001113381	CCL7	Chemokine (C-C motif) ligand 7
F09	XM_001109808	CCL26	C-C motif chemokine 26-like
F10	NM_001047148	LTA	Lymphotoxin alpha (TNF superfamily, member 1)
F11	NM_001032915	MIF	Macrophage migration inhibitory factor (glycosylation-inhibiting factor)
F12	XM_001090562	NAMPT	Nicotinamide phosphoribosyltransferase
G01	XM_001110148	OSM	Oncostatin M
G02	XM_001102788	PF4	Platelet factor 4
G03	XM_001093307	SPP1	Secreted phosphoprotein 1
G04	NM_001047149	TNF	Tumor necrosis factor
G05	XM_001096915	TNFRSF11B	Tumor necrosis factor receptor superfamily, member 11b
G06	XM_001084768	TNFSF10	Tumor necrosis factor (ligand) superfamily, member 10
G07	XM_001092669	TNFSF11	Tumor necrosis factor (ligand) superfamily, member 11
G08	XM_001082247	TNFSF13B	Tumor necrosis factor (ligand) superfamily, member 13b
G09	XM_001089169	TNFSF14	Tumor necrosis factor (ligand) superfamily, member 14
G10	XM_001101190	TNFSF4	Tumor necrosis factor (ligand) superfamily, member 4
G11	XM_001089925	VEGFA	Vascular endothelial growth factor A
G12	NM_001032947	XCL1	Chemokine (C motif) ligand 1
H01	NM_001033084	ACTB	Actin, beta
H02	NM_001047137	B2M	Beta-2-microglobulin
H03	NM_001195426	GAPDH	Glyceraldehyde-3-phosphate dehydrogenase
H04	XM_001097691	LOC70918 6	Hypoxanthine-guanine phosphoribosyltransferase-like
H05	XM_001115079	RPL13A	Ribosomal protein L13A
H06	SA_00125	QGDC	Rhesus Macaque Genomic DNA Contamination
H07	SA_00104	RTC	Reverse Transcription Control
H08	SA_00104	RTC	Reverse Transcription Control
H09	SA_00104	RTC	Reverse Transcription Control
H10	SA_00103	PPC	Positive PCR Control
H11	SA_00103	PPC	Positive PCR Control
H12	SA_00103	PPC	Positive PCR Control

Data analysis setup

Sample management

Control Group	Group 1	Group 2
IR44	FJ69	EP22
IC80	EN60	GH09
JV66	DI62	EP49
JI34		JH54
JD56		

Pre-amplification

A pre-amplification using the appropriate species- and pathway-specific RT^2 PreAMP Primer Mix was not performed and the appropriate corrections were made during the data analysis procedure.

Lower limit of detection

The C_T cut-off was set to 35

Data quality control (QC)

Quality checks performed and results

Test Performed	Test Result
1. PCR Array Reproducibility	All Samples Passed
2. RT Efficiency	All Samples Passed
3. Genomic DNA Contamination	All Samples Passed

Normalization analysis

Manual Selection

Groups	Samples	ACTB	B2M	GAPDH	LOC709186	RPL13A	Arithmetic Mean	Average Arithmetic Mean
Control Group	IR44	19.382416	19.18128	18.76834	21.58224	22.630383	20.31	22.68
Control Group	IC80	19.480572	19.844894	18.954979	22.31814	22.83334	20.69	
Control Group	JV66	25.026503	21.436218	20.55034	24.654093	24.318314	23.20	
Control Group	JI34	25.420666	24.325623	24.366943	25.953773	26.126802	25.24	
Control Group	JD56	24.52721	22.755308	22.292664	25.477743	24.78266	23.97	
Group 1	FJ69	22.210855	21.718853	22.586597	24.846687	26.307396	23.53	22.84
Group 1	EN60	20.71973	20.689894	21.4477	23.195189	24.768885	22.16	
Group 1	DI62	21.84152	21.160624	22.390528	23.10165	25.68201	22.84	
Group 2	EP22	23.707851	23.145807	23.024384	25.433107	27.377485	24.54	23.99
Group 2	GH09	22.53764	21.525318	21.868061	26.136717	26.665062	23.75	
Group 2	EP49	22.73471	21.74011	22.384611	26.689528	26.484142	24.01	
Group 2	JH54	22.261955	22.021616	22.306747	25.860516	25.79681	23.65	

This method allows researchers to select their own internal control / housekeeping / normalization genes for the analysis. Select either the use of the Geometric or Arithmetic Mean consistently across experiments. As a general guide, only select genes with small changes in their expression across different sample groups (differences in C_T values less than 1).

Results

Fold regulation comparison and p-value

Control Group	Test Group	Fold Regulation cut off	p-Value cut off
Control Group	Group 1	2	0.05

Position	Gene Symbol	Fold Regulation	p-Value	Comments
A01	AIMP1	60.62	0.000589	
A02	BMP2	4.90	0.013781	
B06	CSF1	-8.58	0.000622	A
B08	CX3CL1	-6.26	0.009253	A
B11	CXCL12	4.23	0.038022	A
C09	IL10RB	11.93	0.001345	A
D04	IL15RA	5.58	0.002062	A
F02	CXCL8	3.93	0.005739	
F03	CXCR2	2.51	0.042693	
F12	NAMPT	3.72	0.003853	
G03	SPP1	77.25	0.016522	
G06	TNFSF10	36.72	0.000249	A

Control Group	Test Group	Fold Regulation cut off	p-Value cut off
Control Group	Group 2	2	0.05

Position	Gene Symbol	Fold Regulation	p-Value	Comments
A01	AIMP1	52.35	0.000328	
A02	BMP2	5.85	0.018389	
B06	CSF1	-4.28	0.000978	A
B08	CX3CL1	-3.28	0.008666	A
B11	CXCL12	5.17	0.002062	
C09	IL10RB	14.35	0.005420	A
C10	IL11RA	11.79	0.020823	
D04	IL15RA	4.24	0.021867	
D05	IL16	8.23	0.003486	
E12	IL6R	5.96	0.001006	
F07	IL9	9.26	0.010594	
F12	NAMPT	3.20	0.014483	
G06	TNFSF10	40.67	0.000409	A
H05	RPL13A	-2.20	0.038891	

Fold-Change (2^ (- Delta Delta C_T)) is the normalized gene expression (2^(- Delta C_T)) in the Test Sample divided the normalized gene expression (2^ (- Delta C_T)) in the Control Sample. Fold-Regulation represents fold-change results in a biologically meaningful way. Fold-change values greater than one indicates a positive- or an up-regulation, and the fold-regulation is equal to the fold-change. Fold-change values less than one indicate a negative or down-regulation, and the fold-regulation is the negative inverse of the fold-change.

The p values are calculated based on a Student's t-test of the replicate 2^ (- Delta C_T) values for each gene in the control group and treatment groups.

Scatter Plot

Test Group	Control Group	Fold Regulation Threshold
Group 1	Control Group	2

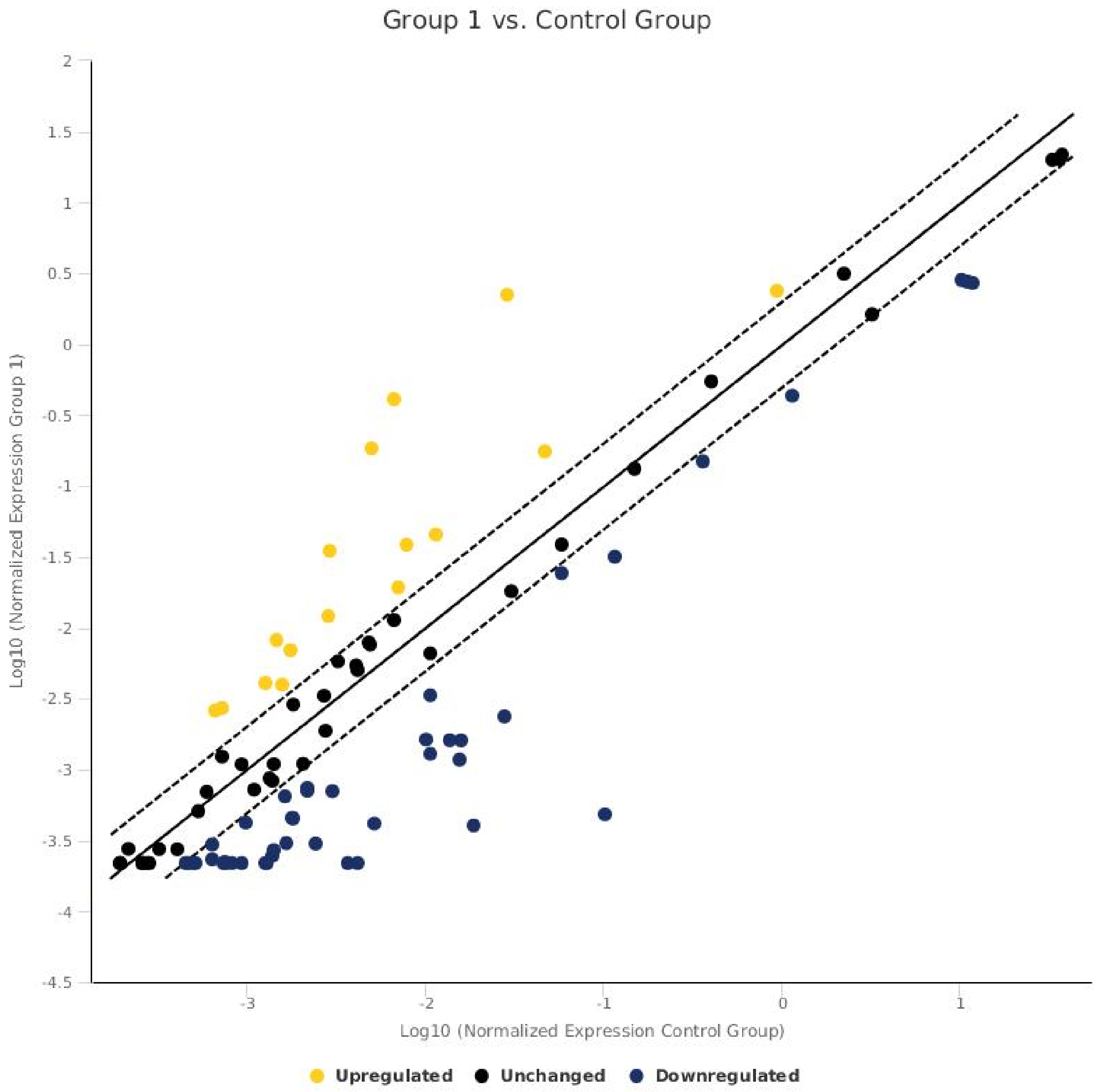

The scatter plot compares the normalized expression of every gene on the array between the two selected groups by plotting them against one another to quickly visualize large gene expression changes. The central line indicates unchanged gene expression. The dotted lines indicate the selected fold regulation threshold. Data points beyond the dotted lines in the upper left and lower right sections meet the selected fold regulation threshold.

Genes Over-Expressed in Group 1 vs. Control Group

Position	Gene Symbol	Fold Regulation	Comments	RT2 Catalog
G03	SPP1	77.25		PPQ08393A
A01	AIMP1	60.62		PPQ01772B
G06	TNFSF10	36.72	A	PPQ01300B
C09	IL10RB	11.93	A	PPQ09121B
D04	IL15RA	5.58	A	PPQ13502B
A02	BMP2	4.90		PPQ17096A
B11	CXCL12	4.23	A	PPQ00230A
F02	CXCL8	3.93		PPQ00298A
E12	IL6R	3.92		PPQ16038B
D05	IL16	3.92	B	PPQ00006A
D01	IL12RB2	3.77	B	PPQ05560B
F12	NAMPT	3.72		PPQ04187A
F07	IL9	3.20	B	PPQ13177A
C10	IL11RA	2.71		PPQ05242A
H01	ACTB	2.53		PPQ00182A
F03	CXCR2	2.51		PPQ05376B

Genes Under-Expressed in Group 1 vs. Control Group

Position	Gene Symbol	Fold Regulation	Comments	RT2 Catalog
G04	TNF	-210.24	A	PPQ15261B
B04	CD40LG	-46.37	A	PPQ00062B
D02	IL13	-19.17	B	PPQ00221A
B07	CSF2	-16.96	B	PPQ00266A
C08	IL10RA	-13.18	A	PPQ08033B
G07	TNFSF11	-12.54	B	PPQ05082B
E10	IL5RA	-11.77	A	PPQ08202B
D08	IL1A	-9.86	A	PPQ05235B
B06	CSF1	-8.58	A	PPQ06801A
D10	IL1R1	-8.30	A	PPQ12806B
B12	CXCL13	-8.17	B	PPQ00124A
B08	CX3CL1	-6.26	A	PPQ00125A
A09	CCL23	-5.90	B	PPQ00261A
F10	LTA	-5.82	B	PPQ15229B
B03	CCR8	-5.68	B	PPQ00839B
C11	IL12B	-5.49	B	PPQ01495B
F08	CCL7	-5.23	B	PPQ14785B
H09	RTC	-4.36		PPX63340A
A05	CCL13	-4.27	B	PPQ14893B
G09	TNFSF14	-4.26	B	PPQ06087A
C12	IL12RB1	-4.01	B	PPQ00099A
H08	RTC	-3.96		PPX63340A
A10	CCL5	-3.91	B	PPQ00080A
F06	LOC710618	-3.74	B	PPQ12114B
G05	TNFRSF11B	-3.63		PPQ06360B
H07	RTC	-3.55		PPX63340A
A12	CCR3	-3.49	B	PPQ13872B
A06	CCL17	-3.38	B	PPQ00082A
F04	IL9R	-3.37	B	PPQ07961B
B10	CXCL11	-3.21	A	PPQ00267A
E08	IL4	-3.10	B	PPQ00178A
D11	IL1R2	-2.97	B	PPQ12748B
D12	IL1RN	-2.74	B	PPQ06231B
F11	MIF	-2.62		PPQ00195A
A03	CCL1	-2.54	B	PPQ00079A
H05	RPL13A	-2.43		PPQ00210B
G08	TNFSF13B	-2.42		PPQ00923B
A04	CCL11	-2.35	B	PPQ00118A
D07	IL17F	-2.32	B	PPQ10562B
C07	IFNG	-2.28	B	PPQ00179A
E01	IL2	-2.17	B	PPQ10428C
C03	CXCR1	-2.16	B	PPQ00429B
E03	IL21	-2.09	B	PPQ10506A

Test Group	Control Group	Fold Regulation Threshold
Group 2	Control Group	2

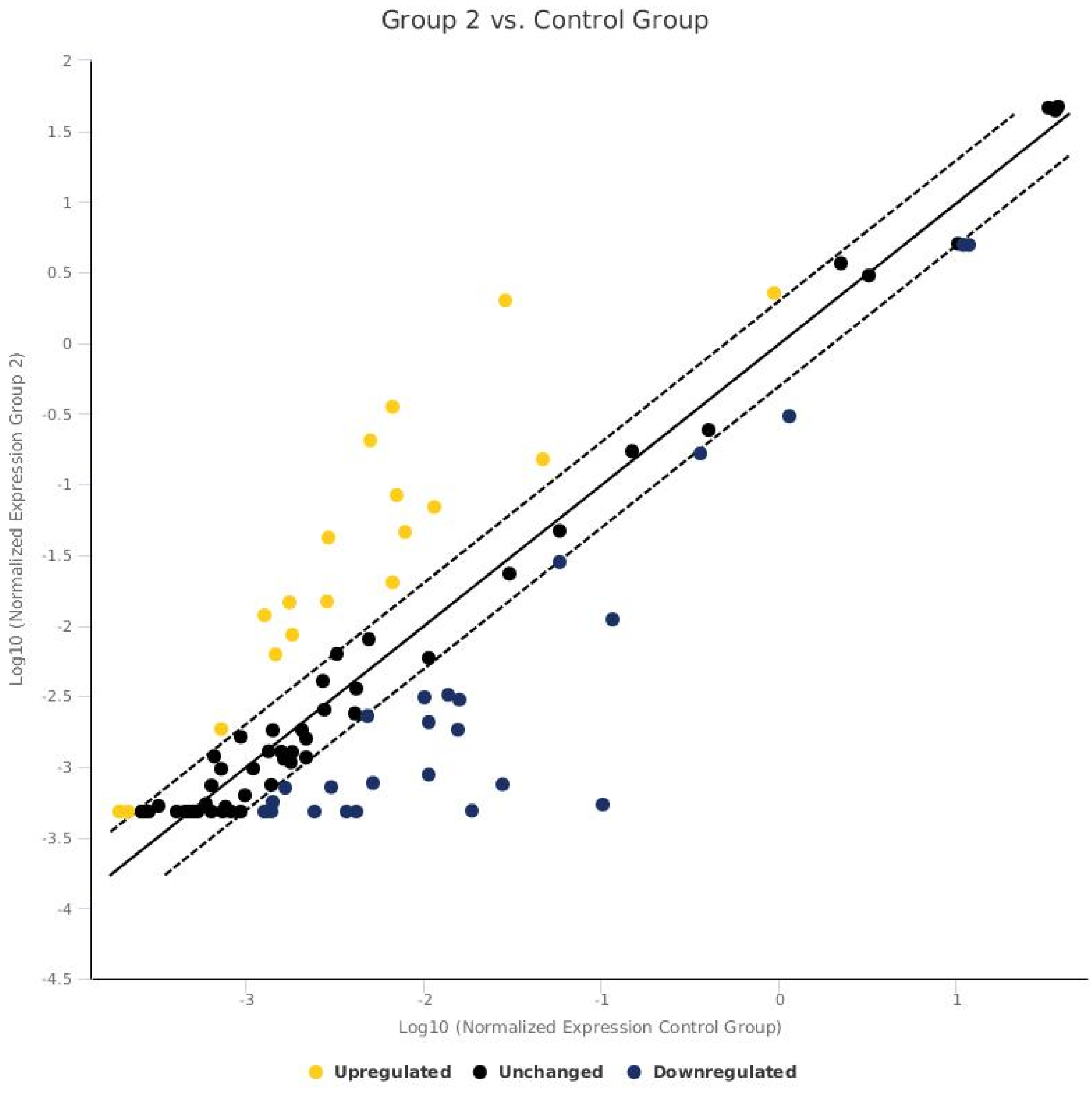

The scatter plot compares the normalized expression of every gene on the array between the two selected groups by plotting them against one another to quickly visualize large gene expression changes. The central line indicates unchanged gene expression. The dotted lines indicate the selected fold regulation threshold. Data points beyond the dotted lines in the upper left and lower right sections meet the selected fold regulation threshold.

Genes Over-Expressed in Group 2 vs. Control Group

Position	Gene Symbol	Fold Regulation	Comments	RT2 Catalog
G03	SPP1	69.35		PPQ08393A
A01	AIMP1	52.35		PPQ01772B
G06	TNFSF10	40.67	A	PPQ01300B
C09	IL10RB	14.35	A	PPQ09121B
C10	IL11RA	11.79		PPQ05242A
F07	IL9	9.26		PPQ13177A
D05	IL16	8.23		PPQ00006A
E12	IL6R	5.96		PPQ16038B
A02	BMP2	5.85		PPQ17096A
B11	CXCL12	5.17		PPQ00230A
A11	CCR1	4.67	B	PPQ00088A
D04	IL15RA	4.24		PPQ13502B
F12	NAMPT	3.20		PPQ04187A
C04	CXCR4	3.04		PPQ09953B
D01	IL12RB2	2.57	B	PPQ05560B
H06	QGDC	2.47	C	
D06	IL17A	2.47	C	PPQ10504B
H01	ACTB	2.40		PPQ00182A
E02	IL20	2.24	B	PPQ01456A

Genes Under-Expressed in Group 2 vs. Control Group

Position	Gene Symbol	Fold Regulation	Comments	RT2 Catalog
G04	TNF	-188.08	A	PPQ15261B
B04	CD40LG	-38.17	A	PPQ00062B
E10	IL5RA	-37.21	A	PPQ08202B
B10	CXCL11	-12.17	A	PPQ00267A
G05	TNFRSF11B	-10.45	A	PPQ06360B
D02	IL13	-8.70	B	PPQ00221A
C08	IL10RA	-8.47	A	PPQ08033B
B07	CSF2	-7.69	B	PPQ00266A
G07	TNFSF11	-6.79	B	PPQ05082B
D08	IL1A	-5.32	A	PPQ05235B
D10	IL1R1	-5.17	A	PPQ12806B
B12	CXCL13	-5.07	B	PPQ00124A
B06	CSF1	-4.28	A	PPQ06801A
A05	CCL13	-4.19	B	PPQ14893B
F11	MIF	-3.74		PPQ00195A
B08	CX3CL1	-3.28	A	PPQ00125A
B03	CCR8	-2.89	B	PPQ00839B
A09	CCL23	-2.68	B	PPQ00261A
F10	LTA	-2.64	B	PPQ15229B
F08	CCL7	-2.51	B	PPQ14785B
H09	RTC	-2.38		PPX63340A
C11	IL12B	-2.34	B	PPQ01495B
H08	RTC	-2.21		PPX63340A
H05	RPL13A	-2.20		PPQ00210B
F05	IL13RA2	-2.09	B	PPQ10644A
G08	TNFSF13B	-2.09		PPQ00923B

Volcano Plot

Test Group	Control Group	Fold Regulation Threshold	p-Value Threshold
Group 1	Control Group	2	0.05

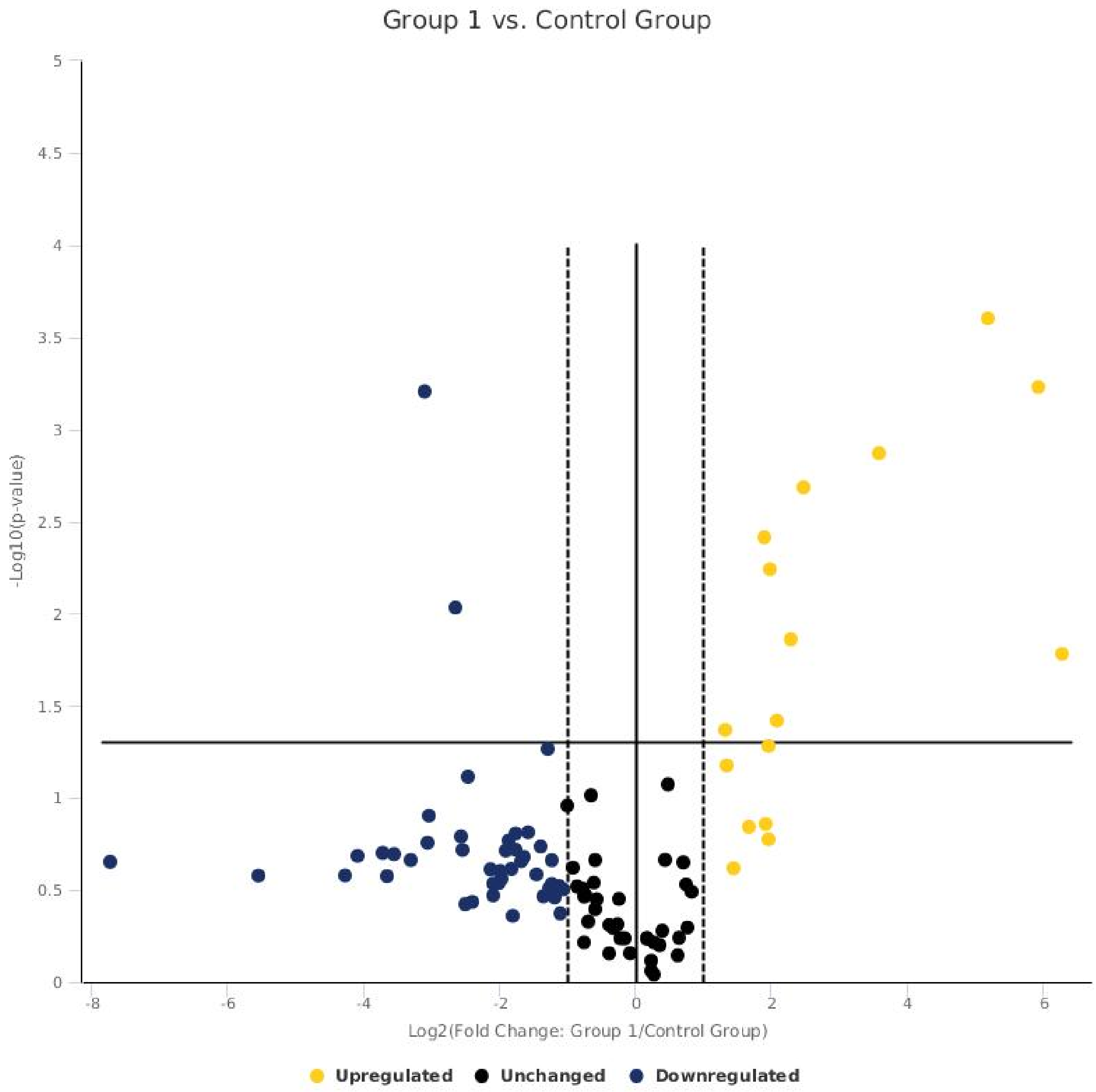

The volcano plot helps quickly identify significant gene expression changes. The volcano plot displays statistical significance versus fold-change on the y- and x-axes, respectively. The volcano plot combines a p-value statistical test with the fold regulation change enabling identification of genes with both large and small expression changes that are statistically significant.

Genes Over-Expressed in Group 1 vs. Control Group

Position	Gene Symbol	Fold Regulation	p-Value	Comments	RT2 Catalog
G03	SPP1	77.25	0.016522		PPQ08393A
A01	AIMP1	60.62	0.000589		PPQ01772B
G06	TNFSF10	36.72	0.000249	A	PPQ01300B
C09	IL10RB	11.93	0.001345	A	PPQ09121B
D04	IL15RA	5.58	0.002062	A	PPQ13502B
A02	BMP2	4.90	0.013781		PPQ17096A
B11	CXCL12	4.23	0.038022	A	PPQ00230A
F02	CXCL8	3.93	0.005739		PPQ00298A
F12	NAMPT	3.72	0.003853		PPQ04187A
F03	CXCR2	2.51	0.042693		PPQ05376B

Genes Under-Expressed in Group 1 vs. Control Group

Position	Gene Symbol	Fold Regulation	p-Value	Comments	RT2 Catalog
B06	CSF1	-8.58	0.000622	A	PPQ06801A
B08	CX3CL1	-6.26	0.009253	A	PPQ00125A

Test Group	Control Group	Fold Regulation Threshold	p-Value Threshold
Group 2	Control Group	2	0.05

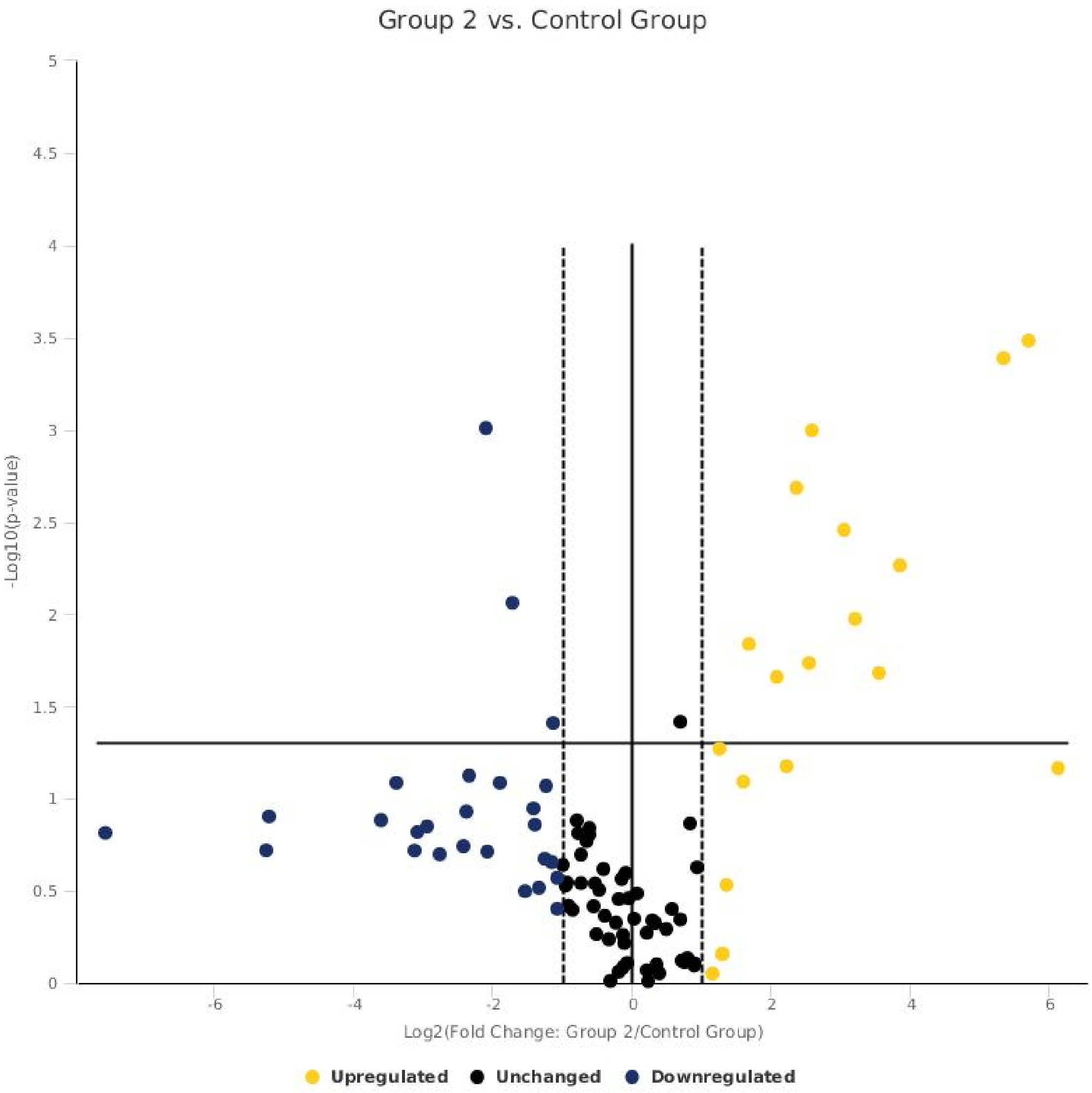

The volcano plot helps quickly identify significant gene expression changes. The volcano plot displays statistical significance versus fold-change on the y- and x-axes, respectively. The volcano plot combines a p-value statistical test with the fold regulation change enabling identification of genes with both large and small expression changes that are statistically significant.

Genes Over-Expressed in Group 2 vs. Control Group

Position	Gene Symbol	Fold Regulation	p-Value	Comments	RT2 Catalog
A01	AIMP1	52.35	0.000328		PPQ01772B
G06	TNFSF10	40.67	0.000409	A	PPQ01300B
C09	IL10RB	14.35	0.005420	A	PPQ09121B
C10	IL11RA	11.79	0.020823		PPQ05242A
F07	IL9	9.26	0.010594		PPQ13177A
D05	IL16	8.23	0.003486		PPQ00006A
E12	IL6R	5.96	0.001006		PPQ16038B
A02	BMP2	5.85	0.018389		PPQ17096A
B11	CXCL12	5.17	0.002062		PPQ00230A
D04	IL15RA	4.24	0.021867		PPQ13502B
F12	NAMPT	3.20	0.014483		PPQ04187A

Genes Under-Expressed in Group 2 vs. Control Group

Position	Gene Symbol	Fold Regulation	p-Value	Comments	RT2 Catalog
B06	CSF1	-4.28	0.000978	A	PPQ06801A
B08	CX3CL1	-3.28	0.008666	A	PPQ00125A
H05	RPL13A	-2.20	0.038891		PPQ00210B

Heat Map

Treated Group	Control Group
Group 1	Control Group

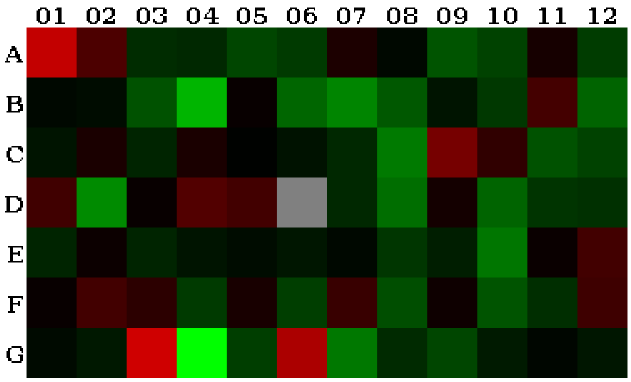

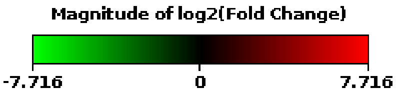

Layout	01	02	03	04	05	06	07	08	09	10	11	12
A	AIMP1 60.62	BMP2 4.90	CCL1 -2.54 B	CCL11 -2.35 B	CCL13 -4.27 B	CCL17 -3.38 B	CCL2 1.77 B	CCL20 -1.19 B	CCL23 -5.90 B	CCL5 -3.91 B	CCR1 1.56 B	CCR3 -3.49 B
B	CCR4 -1.17 B	CCR6 -1.30 B	CCR8 -5.68 B	CD40LG -46.37 A	CD70 1.17 B	CSF1 -8.58 A	CSF2 -16.96 B	CX3CL1 -6.26 A	CX3CR1 -1.51	CXCL11 -3.21 A	CXCL12 4.23 A	CXCL13 -8.17 B
C	CXCL6 -1.53 B	CXCL9 1.69 B	CXCR1 -2.16 B	CXCR4 1.71	FASLG -1.05 B	IFNA2 -1.47 B	IFNG -2.28 B	IL10RA -13.18 A	IL10RB 11.93 A	IL11RA 2.71	IL12B -5.49 B	IL12RB1 -4.01 B
D	IL12RB2 3.77 B	IL13 -19.17 B	IL15 1.20 B	IL15RA 5.58 A	IL16 3.92 B	IL17A 1.12 C	IL17F -2.32 B	IL1A -9.86 A	IL1B 1.53 A	IL1R1 -8.30 A	IL1R2 -2.97 B	IL1RN -2.74 B
E	IL2 -2.17 B	IL20 1.28 B	IL21 -2.09 B	IL2RA -1.50 B	IL2RB -1.31 B	IL2RG -1.56 B	IL3 -1.18 B	IL4 -3.10 B	IL5 -1.88 B	IL5RA -11.77 A	IL6 1.21 B	IL6R 3.92
F	IL7 1.17 B	CXCL8 3.93	CXCR2 2.51	IL9R -3.37 B	IL13RA2 1.64 A	LOC710618 -3.74 B	IL9 3.20 B	CCL7 -5.23 B	CCL26 1.31 B	LTA -5.82 B	MIF -2.62	NAMPT 3.72
G	OSM -1.25 B	PF4 -1.69 B	SPP1 77.25	TNF -210.24 A	TNFRSF11 B -3.63	TNFSF10 36.72 A	TNFSF11 -12.54 B	TNFSF13B -2.42	TNFSF14 -4.26 B	TNFSF4 -1.69	VEGFA -1.12	XCL1 -1.61 A

The Heat Map provides a visualization of the fold changes in expression between the selected groups for every gene in the array in the context of the array layout. The table provides the fold regulation data used for the map as well as the Comments associated with each one.

Treated Group	Control Group
Group 2	Control Group

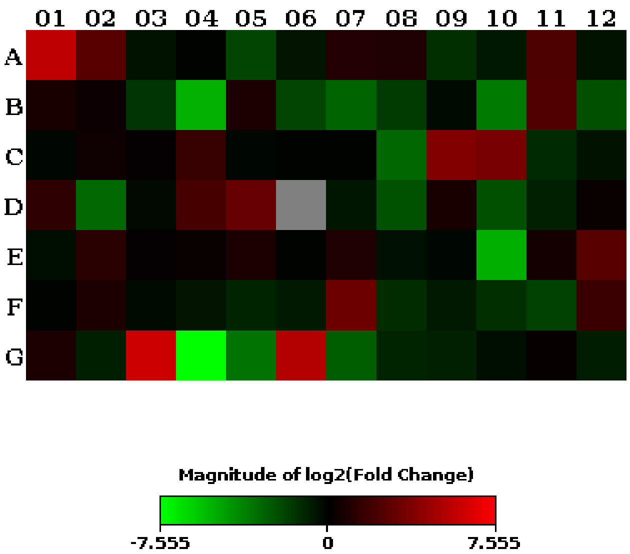

Layout	01	02	03	04	05	06	07	08	09	10	11	12
A	AIMP1<br>52.35	BMP2<br>5.85	CCL1<br>-1.45<br>B	CCL11<br>-1.07<br>B	CCL13<br>-4.19<br>B	CCL17<br>-1.52<br>B	CCL2<br>1.92<br>B	CCL20<br>1.85<br>B	CCL23<br>-2.68<br>B	CCL5<br>-1.66<br>B	CCR1<br>4.67<br>B	CCR3<br>-1.47<br>B
B	CCR4<br>1.65<br>B	CCR6<br>1.28<br>B	CCR8<br>-2.89<br>B	CD40LG<br>-38.17<br>A	CD70<br>1.75<br>B	CSF1<br>-4.28<br>A	CSF2<br>-7.69<br>B	CX3CL1<br>-3.28<br>A	CX3CR1<br>-1.25	CXCL11<br>-12.17<br>A	CXCL12<br>5.17	CXCL13<br>-5.07<br>B
C	CXCL6<br>-1.13<br>B	CXCL9<br>1.32<br>B	CXCR1<br>1.02<br>B	CXCR4<br>3.04	FASLG<br>-1.11<br>B	IFNA2<br>-1.09<br>B	IFNG<br>-1.04<br>B	IL10RA<br>-8.47<br>A	IL10RB<br>14.35<br>A	IL11RA<br>11.79	IL12B<br>-2.34<br>B	IL12RB1<br>-1.43<br>B
D	IL12RB2<br>2.57<br>B	IL13<br>-8.70<br>B	IL15<br>-1.17<br>B	IL15RA<br>4.24	IL16<br>8.23	IL17A<br>2.47<br>C	IL17F<br>-1.56<br>B	IL1A<br>-5.32<br>A	IL1B<br>1.61<br>B	IL1R1<br>-5.17<br>A	IL1R2<br>-1.89<br>B	IL1RN<br>1.16<br>B
E	IL2<br>-1.33<br>B	IL20<br>2.24<br>B	IL21<br>1.05<br>B	IL2RA<br>1.17<br>B	IL2RB<br>1.69<br>B	IL2RG<br>-1.05<br>B	IL3<br>1.86<br>B	IL4<br>-1.39<br>B	IL5<br>-1.14<br>B	IL5RA<br>-37.21<br>A	IL6<br>1.48<br>B	IL6R<br>5.96
F	IL7<br>-1.09<br>B	CXCL8<br>1.78<br>B	CXCR2<br>-1.24<br>B	IL9R<br>-1.53<br>B	IL13RA2<br>-2.09<br>B	LOC7106<br>18<br>-1.70<br>B	IL9<br>9.26	CCL7<br>-2.51<br>B	CCL26<br>-1.73<br>B	LTA<br>-2.64<br>B	MIF<br>-3.74	NAMPT<br>3.20
G	OSM<br>1.76<br>B	PF4<br>-1.89<br>B	SPP1<br>69.35	TNF<br>-188.08<br>A	TNFRSF11<br>B<br>-10.45<br>A	TNFSF10<br>40.67<br>A	TNFSF11<br>-6.79<br>B	TNFSF13B<br>-2.09	TNFSF14<br>-1.93<br>B	TNFSF4<br>-1.31	VEGFA<br>1.15	XCL1<br>-1.81<br>A

The Heat Map provides a visualization of the fold changes in expression between the selected groups for every gene in the array in the context of the array layout. The table provides the fold regulation data used for the map as well as the Comments associated with each one.

ClusterGram

Sample	Dimension	Join Type	Color Coded
Array	2-D	Average	Genes

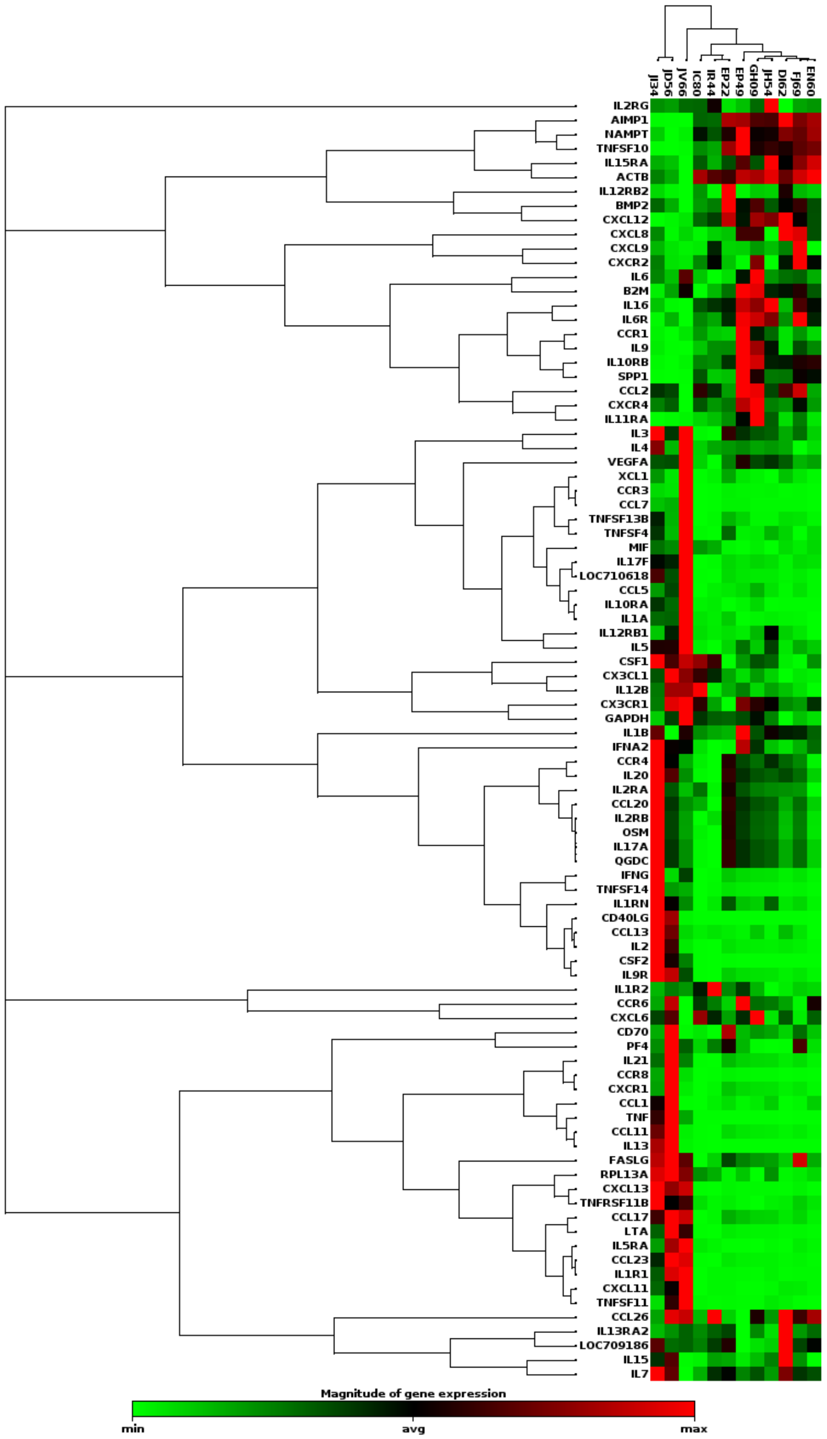

The clustergram performs non-supervised hierarchical clustering of the entire dataset to display a heat map with dendrograms indicating co-regulated genes across groups or individual samples.

What's next?

Thank you for using the RT² Profiler Data Analysis Software.

The Data Analysis software delivers a list of expression changes in the samples from the supplied data. However, this result often only starts an investigation into the underlying mechanisms at work. In order to assist in further analysis, the QIAGEN now utilizes the latest bioinformatics tools to analyze the data and suggest regulatory mechanisms and future experiments. Please review the results from the selected tools below.

Gene Expression: This tool will help define a panel of genes based of this experiment's results. This panel may represent a putative biomarker set, a target gene set or simply a collection of genes. The tool is designed to deliver a list of gene expression assays that would allow the user to follow-up the results of the analyzed experiment.

miRNA Regulation: This tool will identify candidate miRNA regulators in your experimental system. The tool is designed to deliver a list of miRNAs that could be targeting the genes that had observed changes in expression in your selected samples.

DNA Methylation: This tool will help define a panel of differentially expressed genes based on this experiment's results. Altered methylation patterns on the genes' promoters may be responsible for these gene expression changes. This tool is designed to deliver a list of available DNA methylation assays for those differentially expressed genes that would allow the user to follow-up their gene expression experiment with an epigenetic analysis.

Transcription Factor / Histone: This tool will help define a panel of differentially expressed genes based on this experiment's results. Altered transcription factor binding activity on the genes' promoters may be responsible for these gene expression changes. Altered histone modification patterns on the genes' promoters may also be responsible for these gene expression changes. This tool is designed to deliver a list of the transcription factors that might regulate the selected differentially expressed genes as well as the available respective gene-specific real-time PCR assays for DNA from anti-transcription factor or anti-histone chromatin immunoprecipitations. These assays would then allow the user to follow-up their gene expression experiment with an epigenetic analysis.

siRNA: This tool will help define a panel of differentially expressed genes based on this experiment's results. These differentially expressed genes may contribute to the observed differences between the tested sample groups. This tool is designed to deliver a list of differentially expressed genes and corresponding siRNA reagents to test the contribution of each differentially-expressed gene in the experiment. These siRNAs would then allow the user to follow-up their gene expression experiment with a functional analysis.

Somatic Mutation: This tool will help define a panel of genes based on this experiment's results. Mutations in these genes

may affect whether their expression changes have any effect or different effects on the experimental model system. The tool is designed to deliver a list of available somatic mutation assays for these differentially expressed genes that would allow the user to follow-up the results of the analyzed experiment.

Gene Expression, DNA Methylation, RNAi, Somatic Mutation

Test Group	Control Group	Fold Regulation Threshold	p-Value Threshold
Group 1	Control Group	2	0.05

Position	Symbol	Fold Regulation	p-Value	RT2 qPCR Assay	EpiTect Methyl II qPCR Assay	FlexiTube siRNA	Somatic Mutation Assay
G03	SPP1	77.25	0.016522	PPQ08393A	Inquire	View siRNAs	Inquire
A01	AIMP1	60.62	0.000589	PPQ01772B	Inquire	View siRNAs	Inquire
G06	TNFSF10	36.72	0.000249	PPQ01300B	Inquire	View siRNAs	Inquire
C09	IL10RB	11.93	0.001345	PPQ09121B	Inquire	View siRNAs	Inquire
B06	CSF1	-8.58	0.000622	PPQ06801A	Inquire	View siRNAs	Inquire
B08	CX3CL1	-6.26	0.009253	PPQ00125A	Inquire	View siRNAs	Inquire
D04	IL15RA	5.58	0.002062	PPQ13502B	Inquire	View siRNAs	Inquire

Test Group	Control Group	Fold Regulation Threshold	p-Value Threshold
Group 2	Control Group	2	0.05

Position	Symbol	Fold Regulation	p-Value	RT2 qPCR Assay	EpiTect Methyl II qPCR Assay	FlexiTube siRNA	Somatic Mutation Assay
A01	AIMP1	52.35	0.000328	PPQ01772B	Inquire	View siRNAs	Inquire
G06	TNFSF10	40.67	0.000409	PPQ01300B	Inquire	View siRNAs	Inquire
C09	IL10RB	14.35	0.005420	PPQ09121B	Inquire	View siRNAs	Inquire
C10	IL11RA	11.79	0.020823	PPQ05242A	Inquire	View siRNAs	Inquire
F07	IL9	9.26	0.010594	PPQ13177A	Inquire	View siRNAs	Inquire
D05	IL16	8.23	0.003486	PPQ00006A	Inquire	View siRNAs	Inquire
E12	IL6R	5.96	0.001006	PPQ16038B	Inquire	View siRNAs	Inquire
A02	BMP2	5.85	0.018389	PPQ17096A	Inquire	View siRNAs	Inquire
B11	CXCL12	5.17	0.002062	PPQ00230A	Inquire	View siRNAs	Inquire

miRNA Regulation

Test Group	Control Group	Fold Regulation Threshold	p-Value Threshold
Group 1	Control Group	2	0.05

Genes Under-Expressed in Group 1 vs. Control Group

Position	Gene Symbol	Fold Regulation	p-Value
B06	CSF1	-8.58	0.000622
B08	CX3CL1	-6.26	0.009253

miRNA Regulating Genes Under-Expressed in Group 1 vs. Control Group

miRNA Name	Strongest Strength Score	miScript Assay	miScript Mimic	miScript Inhibitor	Target Genes

Genes Over-Expressed in Group 1 vs. Control Group

Position	Gene Symbol	Fold Regulation	p-Value
G03	SPP1	77.25	0.016522
A01	AIMP1	60.62	0.000589
G06	TNFSF10	36.72	0.000249
C09	IL10RB	11.93	0.001345
D04	IL15RA	5.58	0.002062

miRNA Regulating Genes Over-Expressed in Group 1 vs. Control Group

miRNA Name	Strongest Strength Score	miScript Assay	miScript Mimic	miScript Inhibitor	Target Genes

Test Group	Control Group	Fold Regulation Threshold	p-Value Threshold
Group 2	Control Group	2	0.05

Genes Under-Expressed in Group 2 vs. Control Group

Position	Gene Symbol	Fold Regulation	p-Value

miRNA Regulating Genes Under-Expressed in Group 2 vs. Control Group

miRNA Name	Strongest Strength Score	miScript Assay	miScript Mimic	miScript Inhibitor	Target Genes

Genes Over-Expressed in Group 2 vs. Control Group

Position	Gene Symbol	Fold Regulation	p-Value
A01	AIMP1	52.35	0.000328
G06	TNFSF10	40.67	0.000409
C09	IL10RB	14.35	0.005420
C10	IL11RA	11.79	0.020823
F07	IL9	9.26	0.010594
D05	IL16	8.23	0.003486
E12	IL6R	5.96	0.001006
A02	BMP2	5.85	0.018389
B11	CXCL12	5.17	0.002062

miRNA Regulating Genes Over-Expressed in Group 2 vs. Control Group

miRNA Name	Strongest Strength Score	miScript Assay	miScript Mimic	miScript Inhibitor	Target Genes

Transcription Factor / Histone

Test Group	Control Group	Fold Regulation Threshold	p-Value Threshold
Group 1	Control Group	2	0.05

Genes Differentially Expressed in Group 1 vs. Control Group

Position	Gene Symbol	Fold Regulation	p-Value	Transcription Factors	EpiTect ChIP qPCR Assay
G03	SPP1	77.25	0.016522		
A01	AIMP1	60.62	0.000589		
G06	TNFSF10	36.72	0.000249		
C09	IL10RB	11.93	0.001345		
B06	CSF1	-8.58	0.000622		
B08	CX3CL1	-6.26	0.009253		
D04	IL15RA	5.58	0.002062		

Test Group	Control Group	Fold Regulation Threshold	p-Value Threshold
Group 2	Control Group	2	0.05

Genes Differentially Expressed in Group 2 vs. Control Group

Position	Gene Symbol	Fold Regulation	p-Value	Transcription Factors	EpiTect ChIP qPCR Assay
A01	AIMP1	52.35	0.000328		
G06	TNFSF10	40.67	0.000409		
C09	IL10RB	14.35	0.005420		
C10	IL11RA	11.79	0.020823		
F07	IL9	9.26	0.010594		
D05	IL16	8.23	0.003486		
E12	IL6R	5.96	0.001006		
A02	BMP2	5.85	0.018389		
B11	CXCL12	5.17	0.002062		

Next steps

After using QIAGEN's RT^2 Profiler PCR array, use the upregulated or downregulated qPCR assays to further validate your hypothesis.

You can use individual qPCR RT^2 assays or create custom RT^2 PCR arrays.

Further, you can use the assay and other products discussed above in the "What's next?" section to design additional studies on the expression and function of miRNAs regulating the differentially expressed genes, somatic mutations in those genes, epigenetic marks (such as modified histones, transcription factor binding, and DNA methylation) at the promoters of those genes, or study the genes' function using gene-specific siRNA.

Glossary

Comments

A: This gene's average threshold cycle is relatively high (> 30) in either the control or the test sample, and is reasonably low in the other sample (< 30).These data mean that the gene's expression is relatively low in one sample and reasonably detected in the other sample suggesting that the actual fold-change value is at least as large as the calculated and reported fold-change result. This fold-change result may also have greater variations if p value > 0.05; therefore, it is important to have a sufficient number of biological replicates to validate the result for this gene.

B: This gene's average threshold cycle is relatively high (> 30), meaning that its relative expression level is low, in both control and test samples, and the p-value for the fold-change is either unavailable or relatively high (p > 0.05).This fold-change result may also have greater variations; therefore, it is important to have a sufficient number of biological replicates to validate the result for this gene.

C: This gene's average threshold cycle is either not determined or greater than the defined cut-off (default 35), in both samples meaning that its expression was undetected, making this fold-change result erroneous and un-interpretable.
